# Competing Intramolecular *vs.* Intermolecular Hydrogen Bonds in Solution

**DOI:** 10.3390/ijms151119562

**Published:** 2014-10-28

**Authors:** Peter I. Nagy

**Affiliations:** Center for Drug Design and Development, the University of Toledo, Toledo, OH 43606-3390, USA; E-Mail: pnagy@utnet.utoledo.edu; Tel.: +1-419-866-5101

**Keywords:** conformations, tautomerism, aqueous solution, continuum solvent, Monte Carlo, relative free energies, solute dimerization

## Abstract

A hydrogen bond for a local-minimum-energy structure can be identified according to the definition of the International Union of Pure and Applied Chemistry (IUPAC recommendation 2011) or by finding a special bond critical point on the density map of the structure in the framework of the atoms-in-molecules theory. Nonetheless, a given structural conformation may be simply favored by electrostatic interactions. The present review surveys the in-solution competition of the conformations with intramolecular *vs.* intermolecular hydrogen bonds for different types of small organic molecules. In their most stable gas-phase structure, an intramolecular hydrogen bond is possible. In a protic solution, the intramolecular hydrogen bond may disrupt in favor of two solute-solvent intermolecular hydrogen bonds. The balance of the increased internal energy and the stabilizing effect of the solute-solvent interactions regulates the new conformer composition in the liquid phase. The review additionally considers the solvent effects on the stability of simple dimeric systems as revealed from molecular dynamics simulations or on the basis of the calculated potential of mean force curves. Finally, studies of the solvent effects on the type of the intermolecular hydrogen bond (neutral or ionic) in acid-base complexes have been surveyed.

## 1. Introduction

Conformational preferences can cause non-contiguous atoms within an isolated molecule to become similarly close neighbors. These spatial arrangements may be driven by favorable electrostatic interactions or by the special case where three of such atoms form a so-called “hydrogen bond” (H-bond). Although the situation becomes more complicated when the molecular structure is considered within a solution environment, these same two factors remain important to also drive additional intermolecular interactions now possible between solute molecules themselves and with the solvent molecules as partners. Focusing herein on hydrogen bonding, it can be noted that, despite a decades-long endeavor to define the H-bond, this key arrangement still cannot be considered to be resolved with full consensus. The 2011 IUPAC recommendations provide a definition [1] that can be used as the basis for critical evaluation of a number of structural that will be examined in Section 3 of this review. The recommendations state:

“The hydrogen bond is an attractive interaction between a hydrogen atom from a molecule or a molecular fragment *X*–H in which *X* is more electronegative than H, and an atom or a group of atoms in the same or a different molecule, in which there is evidence of bond formation.

A typical hydrogen bond may be depicted as *X*–H…*Y*–*Z*, where the three dots denote the bond. *X*–H represents the hydrogen bond donor. The acceptor may be an atom or an anion *Y*, or a fragment or a molecule *Y*–*Z*, where *Y* is bonded to *Z*. In some cases, *X* and *Y* are the same. In more specific cases, *X* and *Y* are the same and *X*–H and *Y*–H distances are the same as well leading to symmetric hydrogen bonds. In any event, the acceptor is an electron rich region such as, but not limited to, a lone pair of *Y* or π-bonded pair of *Y*–*Z*. The evidence for hydrogen bond formation may be experimental or theoretical, or ideally, a combination of both. Some criteria useful as evidence and some typical characteristics for hydrogen bonding, not necessarily exclusive. The greater the number of criteria satisfied, the more reliable is the characterization as a hydrogen bond.” A footnote (F1) emphasizes that “…there will be borderline cases for which the interpretation of the evidence might be subjective. In any case, there should be no gross deviations from the above-mentioned criteria.”

Six criteria and several characteristics are also included in the article, and eight points, as footnotes, help the reader to interpret the used terms. The first criterion for a hydrogen bond claims: “The forces involved in the formation of a hydrogen bond include those of an electrostatic origin, those arising from charge transfer between the donor and acceptor leading to partial covalent bond formation between H and *Y*, and those originating from dispersion.” It reveals from the specification in footnote F2 that “Attractive interactions arise from electrostatic forces between permanent multipoles, inductive forces between permanent and induced multipoles, and London dispersion forces. If an interaction is primarily due to dispersion forces, then it would not be characterized as a hydrogen bond.”

According to the classification of Grabowski [2], the stabilization energy from weak to moderate hydrogen bonds is 4–63 kJ/mol. The hydrogen bonds in the present survey generally reside in “weak” to at most “moderate” categories. For the molecules analyzed in section 3, the *X* and *Y* atoms are separated by at least two atoms along the covalently bonded substructure path. A basic requirement for the formation of a hydrogen bond is a short distance between the H and *Y* atoms. If the *X* and *Y* atoms are O or N, the *X*–H covalent bond is generally polarized, and the *Y* atom carries an electron lone-pair pointing toward the H atom. Through formation of a H-bond between the indicated atoms, dispersion forces are much less important than the electrostatic interactions and the charge transfers.

In a special case, *Y* symbolizes an aromatic ring with its electron cloud favorably interacting with a positively polarized H atom. This sort of hydrogen bond is called an H…π interaction. For *X*–H…*Y* with *X* = C or with *X*, *Y* = S or P, as well as for the H…π interaction, the role of the dispersion forces increases in comparison to the cases where the H-bond formation is principally related to electrostatic and charge-transfer effects. A situation where the dispersion interactions are apparently important will be discussed as part of the conformational analysis of tyramine in Subsection 3.3.1

The third criterion (E3) on the list of the IUPAC recommendations says: “The *X*–H…*Y* angle is usually linear (180°) and the closer the angle is to 180°, the stronger is the hydrogen bond and the shorter is the H…*Y* distance.” Two important footnotes were added to this criterion. “The *X*–H…*Y* hydrogen bond angle tends toward 180° and should preferably be above 110° (F4).” “Historically, the *X* to *Y* distance was found to be less than the sum of the van der Waals radii of *X* and *Y*, and this shortening of the distance was taken as an infallible indicator of hydrogen bonding. However, this empirical observation is true only for strong hydrogen bonds. This criterion is not recommended. In most cases, the distance between H and *Y* are found to be less than the sum of their van der Waals radii. It should be noted that the experimental distances are vibrational averages and would differ from such distances calculated from potential energy minimization. (F5)”.

**Figure 1:**
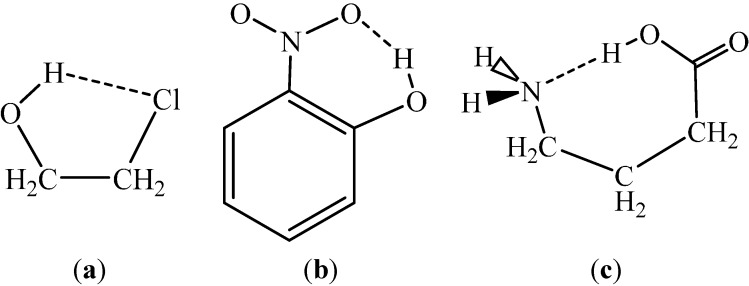
The figure shows the projection of the heavy-atom skeleton onto the *X*–H…*Y* plane for cases where H-bonding can result in a: (**a**) Five-member ring; (**b**) Six-member ring; or (**c**) Seven-member ring.

Thus, as revealed by the quoted text, no H…*Y* or *X*…*Y* distance has been strictly defined for the distance of a H-bond, nor has a strict lower limit for the *X*–H…*Y* angle has. On the other hand, the *X*…*Y* distances for the different intramolecular H-bonds could represent borderline cases with values equal or slightly larger than the sums of the van der Waals radii. Likewise, in cases when a H-bond can form a five-member ring arrangement (Figure 1), the *X*–H…*Y* bond angles could be close to or even less than 110°.

**Figure 2:**
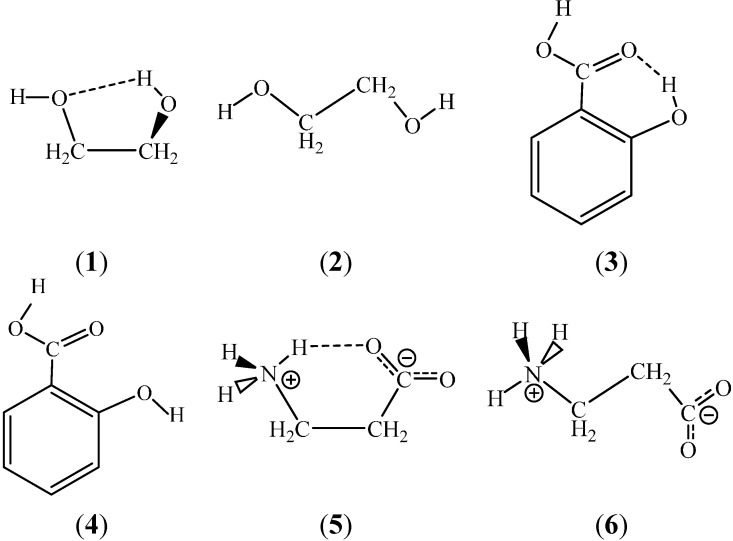
Structures with an intramolecular hydrogen bond for: (**1**) 1,2-Ethanediol; (**2**) Salicylic acid; (**3**) Hydroxy-benzoic acid; and (**5**) β-Alanine zwitterion. Conformations **2**, **4**, **6** prevent the formation of the intramolecular H-bond and are open for forming intermolecular hydrogen bonds.

Another point deserves consideration when qualifying whether a bond meets the criteria of being a H-bond. The atoms-in-molecules (AIM) theory of Bader and Popelier [3,4] identifies the H-bond in a topological manner. The theory can be applied to find bond critical points (BCP) and to analyze them in terms of electron densities and their Laplacian. Qualification is primarily related to the existence of a bond critical point of (3, −1) type, but there are seven more features to be met [5]. One of them is the mutual penetration of the hydrogen and the acceptor atoms. This characteristic of a H-bond guarantees that polar *X*–H and *Y* groups cannot form an intramolecular hydrogen bond if they are far from each other in the space. For a rigid system, e.g., 1,4-dihydroxy benzene, the chemical structure itself prevents the penetration. For saturated chain systems with conformational flexibility, an extended form like the *trans* conformation for the *X*–CH_2_–CH_2_-*Y* moiety prevents the necessary closeness of the polar groups (Figure 2). Consequently, no (3, −1) BCP could be found for the above structures.

The IUPAC recommendations, however, do not include the (3, −1) BCP as a necessary criterion for the existence of a H-bond. Instead, this feature is considered (C6) only a *characteristic* of a H-bond on the basis of theoretical results: “Analysis of the electron density topology of hydrogen-bonded systems usually shows a bond path connecting H and *Y* and a (3, −1) bond critical point between H and *Y*.”

This C6 point in combination with F1 above is very important in understanding several computational results. Klein [6] did not find a (3, −1) BCP regarding a O–H…O intramolecular hydrogen bond for 1,2 diols in their optimized geometries. Mandado *et al.*, [7] found the (3, −1) bond critical point missing for gas-phase 1,2-dihydroxybenzene (catechol) with the geometry optimized at the B3LYP/6-311++G** level. The existence of this BCP would have indicated an O–H…O intramolecular hydrogen bond by the AIM theory. A slight distortion of the optimized catechol geometry, however, led to the appearance of the (3, −1) BCP. Thus, this molecule may present a borderline case for a H-bond (see F1 above).

This characterization of an intramolecular H-bond is largely retained for the case of an intermolecular H-bond with the basic difference that the *X*–H covalent bond and the *Y* atom (aromatic ring) are elements of two different molecules. In this case, the two species have to approach each other appropriately in space. Thus, whereas the intramolecular H-bond is a feature of a single molecule in a favorable conformation, the intermolecular H-bond between two molecules emerges only within a specific H…*Y* distance range. Accordingly, H-bond qualification at a separation corresponding to the sum of the van der Waals radii again becomes problematic. Regarding the *X*–H…*Y* bond angle, the values for an intramolecular and intermolecular bond could differ considerably. For the latter, calculations predict a slightly bent H-bond of about 160°–180° in the gas-phase unless there is an additional geometric constraint. The computational result is reasonable: the geometry optimization seeks a structure with minimized strain between the two species. On this basis, the favorable *X*–H…*Y* arrangement is close to linear. This conclusion refers only for isolated pairs, mostly existing in the gas phase. Alternatively, the crystalline phase environment can strongly affect the H-bond geometry [8].

Thus some points above suggest that borderline cases are conceivable, whereas no distance limit was provided in the IUPAC definition. On the other hand, consideration of the sum of the van der Waals radii, as an upper limit is not recommended. Indeed, what are the relevant van der Waals radii? Bondi presented a table for mean values [9], but are these values always relevant in any molecular environment? Could there be a H-bond if the sum of the considered van der Waals radii is almost equal to the H…*Y* distance?

These problems (and probably a number of others) underscore that the definition for a H-bond is not a closed chapter within the field of theoretical chemical research. Recently, Weinhold and Klein [10] published a paper with a detailed list of the former theoretical activities that have been directed toward this topic. The authors proposed a new H-bond definition in partial agreement with the present IUPAC recommendations. It is noteworthy that a topological requirement was also not put forward.

In a recent paper by Contreras-García *et al.* [11], the authors note that the density values at the H-bond critical point cannot be used to identify the most stable geometry of a complex. This statement is in accord with the results from calculations performed by Klein and Mandado *et al.* [6,7], although the latter also found that a (3, −1) BCP could be identified for 1,2-dihydroxy benzene upon a small geometry distortion, which suggests that the optimized and intramolecularly H-bonded structures are not too different. For a more effective analysis of non-covalent interactions, Contreras-García *et al.* [11] developed a new index (non-covalent interactions, NCI) by utilizing the reduced density gradient. Although the method is related to the AIM approach, the NCI features are tied to the critical points of the density gradient field. Use of the reduced density gradient facilitates the account for local density inhomogenities. The NCI isosurfaces reveal the connections of the critical points in the real space, which can form superbasins. Accordingly, as the authors claim, “ring or cage points are sometimes a better reference for understanding bond strength than bond point themselves.”

Then the question remained in this review is: how to identify a hydrogen bond? The problem is more sensitive regarding the formation of an intramolecular H-bond because computations indicate that the (3, −1) BCP can generally be found for intermolecular hydrogen bonds. An important example is the microsolvation of a reference molecule by a few protic solvent molecules when the latter form a bridge within the intramolecular *X*–H…*Y* region [6,12,13,14,15,16]. Water and methanol can arrange in a manner that even one solvent molecule creates two intermolecular hydrogen bonds in the standard form of O–H…*Y* and *X*–H…O. In both arrangements, the H-bond distances can be of favorable length and the bond angles for the newly formed hydrogen bonds can be much closer to 180° than that for the unsolvated reference “solute”. Corresponding (3, −1) BCP’s have been found for the 1,2-ethanediol monohydrate [6] and for the 2,2,2-trifluoroethanol:water 1:1 complexes [16].

Intramolecular H-bonds in the gas phase will be accepted in this review on the basis of experimental studies. For distance considerations, the values provided by Grabowski [2] will be utilized. The *X*…*Y* donor-acceptor separations for strong H-bonds with energy of 63–167 kJ/mol were accepted by Grabowski as 240–255 pm for O–H…O systems, 250–260 pm for the N–H…O bonds and 260–270 pm for the N–H…N interactions. No range was provided for the important O–H…N bonds, for which a characteristic distance of about 260 pm has been estimated here. The H-bonds in the present survey generally fall into the category “moderate”. Accordingly, the *X*…*Y* distances can be assumed to be at somewhat larger values than provided by Grabowski.

An important experimental feature of a H-bond is the shift of the *X*–H stretching frequency. In the case of “proper” hydrogen bonds, the frequency decreases and is called a red shift. Most of the H-bonds belong to this category [17]. In some cases, however, the *X*–H frequency increases upon H-bond formation. This phenomenon is called a “blue” or “improper shift”. The quantum mechanical comparison and the related explanation were summarized by Hobza and Havlas [18].

The shift in stretching frequency is related to the increase and the decrease of the *X*–H bond length in cases of the red- and blue-shifts, respectively. The change of *X*–H bond length is related to a charge transfer from the acceptor to the donor molecule in the H-bonded complex, which can be ascertained by means of NBO analyses [19]. In the case of the red-shift, some charge is transferred from the lone pair of the Y atom to the antibonding *X*–H orbital of the donor molecule. As a result, the *X*–H bond length increases and its stretching frequency decreases. Alternatively, blue-shift was noticed, for the Cl^−^…H_3_CBr system, as well as others. In this complex, the charge is transferred from the anionic acceptor to the antibonding orbital of the C–Br bond. The C–Br bond elongates followed by a geometry reorganization of the H_3_CBr molecule. In its new geometry, the C–H bond becomes shorter providing the basis for the frequency increase and hence the blue-shift in the spectrum. An important point of the review by Hobza and Havlas is the discussion of the (3, −1) BCP’s, which were found both for the proper and improper intermolecular hydrogen bonds. Other AIM criteria for a H-bond were also met for the systems exhibiting blue-shift of the vibrational frequency.

For many systems studied below, AIM analyses were not found during literature searching. Furthermore, even when such calculations are performed, there remains the possibility of not finding a (3, −1) BCP, as happened to the optimized geometry of 1,2-ethanediol [6,7]. Thus the present author does not signify a H-bond to be present or not present based upon the existence of a BCP. This stance is supported by the allowable borderline systems in the IUPAC definition and by the conclusions from the NCI analyses [11] regarding energy-minimized structures. The existence of an intramolecular H-bond will be accepted if the experimentally derived H…*Y* distance is smaller than the sum of the van der Waals radii and/or a meaningful shift in the *X*–H stretching frequency was experimentally recorded.

For a number of isolated molecules, experiments predict (*X*) H…*Y* separations within the 200–250 pm range, with van der Waals radii of 120, 155, and 152 pm for H, N, and O, respectively [9]. For five-member saturated rings (Figure 1), the conformation corresponds to a *X*–C–C–*Y* gauche arrangement. Even if a (3, −1) BCP is missing, it is conspicuous that this conformation is the most stable one for many molecules in the gas phase. The aim of this review then becomes to consider the solvent effect on the maintenance or modification of the experimentally found gas-phase conformation while leaving the possibility open for forming an intramolecular H-bond. A *gauche* to *trans* transformation of the *X*–CH_2_–CH_2_–*Y* moiety would definitely disrupt an intramolecular H-bond (Figure 2). The intramolecular H-bond also is disrupted upon rotation of 180° about the C–O bond for species **3**.

**Figure 3:**
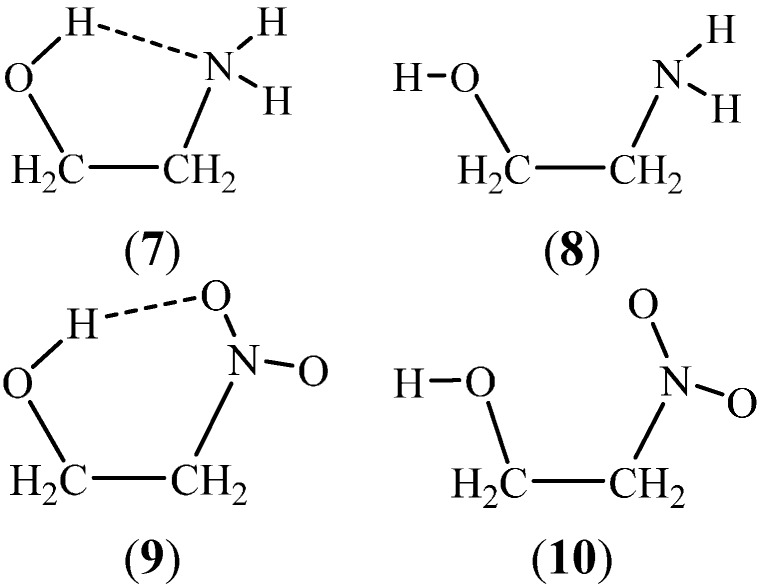
OCCN *gauche* structures with an intramolecular H-bond for 2-aminoethanol (**7**) and 2-NO_2_ ethanol (**9**); Conformations **8** and **10** indicate disrupted H-bonds after rotations by approximately 120° about the O–C axes.

Even if the *gauche* structure for the XCCY moiety is maintained, the intramolecular H-bond associated with a H–*X*–C–C *gauche* arrangement would be disrupted upon rotation about the *X*–C bond resulting in a H–*X*–C–C *trans* conformation (Figure 3). In the case of a six-member intramolecular H-bond, like for the *ortho* substituted phenols in Figure 1, the H-bond is disrupted upon an 180° rotation about the C–O bond.

In aqueous solutions, the O (solute)…O (water) and N (solute)…O (water) radial distribution functions show their first minima at up to 350 pm [20,21]. This value has been accepted as the boundary of the first hydration shell around the polar sites of solutes. This, however, does not mean that intermolecular H-bonds would be expected with *X* (solute)…O (water) separation up to 350 pm in solution. Analyses of Monte Carlo results (see below) always point out that the number of the solvent molecules engaged in H-bond(s) to the solute is smaller than the total number of the solvent molecules in the first hydration shell(s) around the polar site(s). The solute-solvent pair-energy distribution functions show, in general, a maximum and a minimum within the range of −70 to about −10 kJ/mol for aqueous solutions. Integration of this distribution function up to its first minimum was interpreted by Jorgensen *et al.*, [20] as the number of the intermolecular, solute-solvent hydrogen bonds in water.

A recent review by Nagy [22] dealt with the in-solution conformational/tautomeric equilibria for small molecules in general, and the theoretical methods applied in the corresponding calculations were shortly characterized in that review. References to the theoretical methods will be only given for some less-known methods in the present paper. Basis sets applied in quantum mechanical calculations will be provided in cases where they may be needed to evaluate the relevance of the obtained results. Regarding structural analyses, recent publications were mostly sought with the hope that meaningful former studies would be listed in the later ones.

As stated in the title, the present survey emphasizes a special structural problem. Regarding the methodology, only problems related to the modeling of the H-bond will be discussed here. The conformational issue will be investigated for a number of families of small molecules. Unusual structures will not be discussed due to the length-limitations of this paper.

## 2. Methodology

### 2.1. Experimental Methods

As discussed above, the existence of an intramolecular H-bond was accepted in this review on the basis of gas-phase experiments. In a number of the studies the spectra were recorded for jet-cooled systems. References to these are provided when the gas-phase and in-solution molecular structures are compared in Section 3. The jet-cooling technique [23] allows reaching local temperatures as low as about 5 K, with the advantage that the molecules assume their vibrational ground states. Under these conditions, the vibrational spectrum becomes simpler and different conformers can be more easily identified and characterized. Microwave spectra were recorded at room temperature or somewhat below [24,25,26,27]. Gas-phase electron diffraction structure determinations, sometimes at two or three different temperatures, were performed in the range of 297–733 K [28,29,30,31].

For in-solution IR spectra, the temperature was generally room temperature or not far from it in the experiments surveyed here. In these cases, the boiling point of the solvent imposes a limit for the upper temperature. It is known that signals can split in NMR studies by lowering the temperature. The lowest operational temperature is constrained, however, by the freezing point of the NMR solvent [32].

In summary, the gas-phase structural parameters were obtained from experiments conducted in a very large temperature range of 5–733 K. The in-solution investigations were mainly performed near room temperature. Thus, comparisons of the structural data between gas-phase and in-solution experiments, as well as to theoretical calculations referring to 0 K, need caution.

### 2.2. Geometry Optimization

#### 2.2.1. General Problems

The first step in a quantum-chemical structure and energy analysis is the optimization of the molecular geometry. If more than one structural form (different conformers, tautomers) are to be considered, each of them has to be optimized. A very important point is the selection of a reliable theoretical method and the application of a satisfactorily large basis set. Clearly, one wants to obtain the best computational results possible within the technical limits of the given structural problem. When a seeming H-bonded system is under scrutiny, an additional problem emerges in that the system has to be identified whether it is really a hydrogen-bonding arrangement or not.

In the review [22], a reference list was provided for the theoretical approaches and basis sets most frequently used in the past 15–20 years for geometry optimizations and relative energy calculations for ground state, closed-shell systems. Geometries were mainly optimized at the *ab initio* Hartree-Fock (HF) and second-order Møller-Plesset perturbation theory (MP2) levels or using some DFT-based (density functional theory) method such as B3LYP or some more recent ones that account for the dispersion interaction like the B97D method of Grimme [33] or the M05 and M06 methods of Zhao and Truhlar [34]. For basis sets, the 6-311++G** Pople basis or correlation consistent basis sets like cc-pvXz or aug-cc-pvXz (*X* = d, t, q) [35] have been applied more often in the most recent studies. Relative energies of conformers and tautomers from *ab initio* calculations are sensitive to the applied level of theory (method + basis set). Accounting for the electron correlations beyond the MP2 approach has turned out to be very effective by the application of the coupled-cluster methods, CCSD and CCSD(T) (coupled-cluster singles and doubles and noniterative triples) [36,37]. Hobza proposed an extrapolation formula for calculating the molecular energy at the CBS (complete basis set level) utilizing the MP2 limit energy and the difference of the CCSD or CCSD(T) energy and the MP2 energy calculated with some smaller basis set [38]. Frequency analysis can verify local energy minimum geometries by finding all positive vibrational frequencies. Using classical partition functions for ideal-gas molecules, the free energies can be estimated at some temperature *T* and pressure p [39].

While many papers have proven that the results are very sensitive to the level of theory, calculations applying high-level theoretical methods in combination with large basis sets may not be practical even for the case of small molecules. This is true not only for individual geometric parameters and energy values where it is normal that energy decreases with a higher-level method and/or a larger basis set, but even for the relative energies between conformations. Changes in the relative values suggest that the energy differences have not reached a converged limit value yet. A disappointing example was presented by DePrince and Mazziotti [40], who compared two tautomers of the CH_3_NO molecule at the CCSD and CCSD(T) levels. Whereas the relative energies were calculated at 29.1 and 21.4 kJ/mol, respectively, utilizing the cc-pvdz basis set, the CBS values are 2.8 and −6.2 kJ/mol. These changes are dramatic. They indicate that the basis set effect on the relative energy is very large and the selected methods at the CBS limit even predict different relative stabilities. The CCSD(T)/CBS result is perhaps more reliable, yet it can not be ascertained in the absence of experimental information whether the obtained value is an acceptable limit or even higher-level methods should be considered. Note that this problem can be noticed even for a very small molecule. For a larger molecule (e.g., 15–20 heavy atoms and corresponding hydrogens) upgrading the level of theory is even less practical. The situation could be worse regarding the optimized geometries. For relative energies, we can ascertain at least that the computed limit is questionable while for geometries there is no clue about the correct bond lengths, angles, and torsion angles. If they do not vary monotonically in parallel with the increasing level of theory, one may have even less idea about the correct limit values in the absence of experimental information.

Why are the above, otherwise well-known computational experiences important in a review regarding H-bonds? As was shown above, the critical point can emerge after a small distortion of the optimized 1,2-dihydroxybenzene geometry. This finding can be interpreted to mean that the H-bond is disrupted in the optimal geometry. Indeed, it is quite possible that the existence of the BCP is very sensitive to the structure as can be seen by the earlier the quoted notes of Contreras-García *et al.* [11]. Thus, the suspicion may emerge that the level of theory is not high enough when small geometric changes can create or perturb a H-bond. Using again the paper of Mandado *et al.*, [7] as an example, the (3, −1) BCP was found on the B3LYP/6-31+G** density map, but the BCP disappeared at the B3LYP/6-311++G** level, and did not appear either when the B3LYP/6-311++G (3d,3p) density was studied. The authors considered the B3LYP/6-31+G** result as an artifact and attributed it to the lack of diffuse functions on the hydrogen atoms. Disappearance of the BCP with larger basis sets clearly indicates, however, the basis set effect on some calculated topological indeces, and calls for studying a reliable electron density map. Obtainment of the latter is perhaps possible at a very high theoretical level, but such calculations are not practical for larger molecules. On the other hand, if gradually increasing basis sets provide different predictions with respect to the existence of a BCP, then conclusions based on medium-size basis sets remain uncertain.

Theoretical calculations are able to predict a shift in the *X*–H vibrational frequency if the bond is involved H-bond interaction. Gu *et al.*, [41] studied the possible intramolecular H-bond for α-hydroxy acetic acid. The authors concluded that the red-shift of about 100 cm^−1^, based on former experimental results for the stretching frequency of the α-hydroxy group relative to that for a free O–H bond in methanol “can be attributed to internal OH…O= hydrogen bonding”.

The red-shift in the case of an intramolecular H-bond was also demonstrated, at least qualitatively, by HF/6-31G* calculations for the 1,2-ethanediol [42,43]. For the all-*trans*-OCCO conformer tTt (C_2*h*_ symmetry, for the three-letter code see [44]), where the two oxygens are far from each other, the two O–H frequencies were calculated equally at 4124 cm^−1^. For the most stable OCCO *gauche* conformation, tG^+^g^−^ (C_1_ symmetry) allowing for an intramolecular hydrogen bond, the two O–H frequencies differed in accord with the experimental finding (see below). The frequencies for the *gauche* form were calculated at 4095 and 4123 cm^−1^. The smaller value refers to the O–H vibration involved in intramolecular O–H…O bonding. The larger frequency is related to the free OH vibration in the tG^+^g^−^ conformation, where “t” indicates the *trans* HOCC arrangement. As a free OH, its stretching frequency is practically not affected and is equal to that for the tTt conformer. The O…O and O…H distances are 277 and 236 pm, respectively, well within the structural parameter set accepted for a H-bond. Although the calculated high frequencies are generally overestimated by about 10% at the HF level in comparison with experimental values, the shift of the frequency for the O–H group involved in the intramolecular interaction has revealed. A similar conclusion can be drawn for the 2-OH benzoic acid, when the calculated phenolic O–H frequency in intramolecular interaction with the carbonyl oxygen is compared with the free OH stretching frequency, as 3952 *vs.* 4112 cm^−1^ [45].

Florio *et al.*, [46] compared the OH stretching frequencies of the monomeric and dimeric forms of formic and benzoic acids. The experimental values showed a red-shift of 459 cm^−1^ upon formic acid dimerization. The calculated harmonic frequency differences were 556 and 435 cm^−1^ at the B3LYP/aug-cc-pvtz and MP2/aug-cc-pvtz levels, respectively. For formic acid and its dimer, the geometry optimizations by the two methods led to very similar structure parameters, generally also close to the experimental values. The predicted frequencies at the MP2/aug-cc-pvtz level showed consistent overestimations for the monomer and the formic acid dimer (FAD), resulting in a red-shift close to the experimentally observed value. B3LYP/aug-cc-pvtz calculations provided, however, a larger overestimation for the monomer than for the dimer, leading to an increased red-shift. The authors concluded that the results “provide strong evidence that the B3LYP method does not provide a quantitatively correct description of this aspect of the H-bonding in the FAD dimer”.

Upon the benzoic acid dimerization, the red-shift was 217 cm^−1^ experimentally as compared with the theoretical value of 616 cm^−1^ calculated at the B3LYP/6-311+G(2d,2p)/B3LYP/6-31+G(d) level. In this case the red-shift was even more strongly overestimated than that for FAD (see above) by the B3LYP method. These calculations utilized, however, a smaller basis set, B3LYP/6-311+G(2d,2p) for the carboxylic group and 6-31+G(d) for the atoms of the phenyl rings. For the benzoic acid systems, the calculated frequency was overestimated for the monomer and underestimated for the dimer. This latter result differs from that for the formic acid dimer. The calculated large red-shift for the benzoic acid systems may be explained by the interplay of the method and basis set. Since it was already qualified by the authors that the method does not quantitatively describe the H-bond for the dimer of a simple carboxylic acid, this likely applies to the benzoic acid dimer as well.

In intramolecular hydrogen bonds, the geometry for both the H…*Y* distance and the *X*–H…*Y* angle is primarily determined by the covalent structure of the molecule. While three-atom hydrogen-bonded rings are extremely rare, the four-atom substructures (e.g., carboxylic group, amides) deserve special consideration. In most cases, a H-bond can be expected if the system can form a five to seven-member ring, including arrangements utilizing a polar H. Prototypes are indicated in Figure 1 and typical representatives of five- and six-member rings are shown Figure 2 and Figure 3. Seven-member rings can be formed for γ-substituted carboxylic acids, 1,4-disubstituted butanes with OH and/or NH_2_ substituents.

Larger rings are probably not stable. Chen *et al.*, [47] pointed out that no intramolecular H-bond exists in the prevailing conformer of 1,5-pentadiol and 1,6-hexadiol at room temperature. In these cases, formation of an intramolecular H-bond would require a ring conformation with eight and nine members, respectively. This is probably unfavorable due to entropy considerations even for seven-member rings. Nagy *et al.*, [48] studied different conformers for γ-hydroxy-butyric acid. Although the lowest-energy conformer optimized at the MP2/6-311++G** level formed a seven-member ring with an O–H…O= H-bond, the free energy for this structure is higher by about 2 kJ/mol than that for the most stable gas-phase species where this bond is disrupted, as also found experimentally [49]. The results for the increased relative free energy suggest unfavorable entropy effects for the hydrogen-bonded seven-member ring. This explanation is supported by the argument of Blanco *et al.* [50], who investigated the gas-phase structure of γ-amino-butyric acid (GABA). Intramolecular H-bonds were noticed in both forms of N–H…O=C and N…H–O–C=O, although the two mostly populated species do not possess an intramolecular H-bond. In order to create such bonds, structures have to be formed which “contribute to decrease entropy and to increase the Gibbs energy” [50].

#### 2.2.2. Special Problems

In general, for the past twenty years optimized molecular geometries in solution have been obtained by applying a continuum solvent model. The idea was introduced and subsequently developed by the Tomasi group as the PCM model [51,52]. Since the 1990’s, different continuum solvent models [53,54,55,56,57,58,59] and extension beyond the dielectric approximation [60] have been developed to account for the solvent effects on the geometry and energy/free energy of dissolved molecules. Several reviews summarize these models and compare the results obtained from different approaches [52,61,62,63].

The basic idea in the widely used PCM method [51] is that the solute is placed in a cavity carved in the continuum dielectric solvent, and the solute and the solvent mutually polarize each other. As a consequence, the solute’s geometry changes slightly and its internal energy increases when compared to its optimized gas-phase energy. The energy-increase is balanced by the developing solute-solvent electrostatic interaction energy. The final results are obtained through an iterative self-consistent-field (SCF) process that finds the total energy minimum and its related geometry. For the geometry optimization and energy/free energy calculations, all methods can be utilized, which were mentioned in relation to gas-phase calculations [33,34,35,36,37,38]. Thus, geometry optimizations can be performed by means of the HF, MP2 and DFT methods, and higher level energy calculations can be performed up to the CCSD(T)/CBS level. The customary basis set for geometry optimization and frequency analysis is 6-311++G**, but even the aug-cc-pvtz set has been applied [15,64].

When a molecule dissolves, a close molecular environment is encountered that is in contrast to the most frequently applied ideal-gas model, where no potential energy interaction is considered even through the collisions of the molecules. Although the solute-solvent interactions are substantial, the effect of a non-polar or only slightly polar solvent (CCl_4_, CHCl_3_) on the molecular geometry is generally small [15,64]. The geometric effect could be, however, large when a solute with an intramolecular H-bond in the gas phase dissolves in a protic solvent such as water or methanol, which have both proton donor and acceptor sites. In this case, the *X*–H…*Y* intramolecular H-bond may collapse while solute-solvent H-bonds are formed using the free *X*H and *Y* sites.

The weakest point of the continuum dielectric solvent model is that the above solute-solvent H-bond(s) are only implicitly mimicked by polarization of the solvent and concomitant appearance of surface charges on the inner surface of the cavity: Negative surface charges opposite to a polar hydrogen and positive ones in the lone-pair regions of the solute’s oxygens and nitrogens. Although this response is qualitatively correct, the calculated solute-solvent stabilization energy is underestimated [65,66]. Thus, for proper calculation of the free energy changes when a polar solute with or without internal H-bond(s) dissolves in a protic solvent, explicit consideration of the solute-solvent intermolecular H-bonds becomes necessary.

This requirement can be largely satisfied by adopting the supermolecule + continuum approach, where the solute is surrounded by a number of explicit solvent molecules. The solute and the explicit solvent molecules mimic the H-bonds in the first solvation shell within the cavity carved in the continuum solvent. The critical question then becomes, how many explicit solvent molecules are to be considered.

For constructing the starting geometry of a supermolecule, knowledge of microsolvated solute structures is very helpful. In these systems, the central, polar molecule with or without an intramolecular H-bond is solvated by a few solvent molecules. Locations of the solvent molecules (water, methanol) indicate the most preferable solvation sites of the solute with a hydrogen donor/acceptor solvent. Useful information can be obtained from experimental gas-phase hydration/solvation studies augmented with theoretical calculations [12,13,14,16,23,67,68,69,70] or specific theoretical calculations for hydrated amino acid side chains, nucleotid base and sugar models [71,72,73,74,75].

Recent calculations proved [15,64] that application of at least the aug-cc-pvtz basis set is required for reliable estimation of the relative solute free energies. If the solute has 6–10 C, N, O atoms and connected hydrogens, 500 basis functions could easily be required. If such a solute has to be surrounded by at least 5–6 water molecules, the number of basis functions increases to about 1000. The number of basis functions could be somewhat reduced by considering the solvent molecules with a lower basis set, with, e.g., 6-31+G**. While the supermolecule + continuum approach can be useful theoretically, it suffers from several technical challenges.

(1) The geometry optimization for a system with 500–1000 basis functions is very slow in solution. If one wants to prove the local-energy-minimum character of the supermolecule and calculate thermal corrections, very small remaining forces should be allowed only at the end of the optimization. It is almost unreachable for a number of systems (or only by the application of the very time-consuming analytical second-derivative methods), in cases when torsion or intermolecular vibration frequencies could be as low as a few cm^−1^.

(2) The number of explicit solvent molecules to be considered can become be critical. In a real, dilute solution the solute is surrounded by solvent molecules all around. Except for the simplest modeling cases like the partial solvation of γ-amino-butyric acid with 2–5 water molecules [76] or consideration of 3–8 water molecules during the HOCl catalyzed tautomerization of β-cyclopentadione [77], a considerably larger number of water molecules is generally required for reasonable modeling of the solvation sphere even for a small organic molecule. An impressive example was provided by Lu [78], who optimized the geometry of the Al(H_2_O)_6_^3+^·12 H_2_O hydrate at the B3LYP/6-31+G(d,p) level in a water continuum by the PCM method. The resulting structure was of nearly spherical symmetry, easily allowing for the formation of the water network. Consideration of eighteen solvent molecules was necessary for mimicking the first and second hydration shells in a dilute solution.

(3) The results of Lu and coworkers call attention to the need for the supermolecule to reasonably mimic the immediate in-solution environment of the solute. With a relatively small number of explicit solvent molecules within the supermolecule (for example, 3–4 water molecules, originally each of them facing a polar site), the water-water interactions may dominate over the solute-water interactions. Instead of forming 3–4 solute-water hydrogen bonds, a water cluster is then formed on some side of the solute and the number of water-solute hydrogen bonds would be smaller than expected in a water box with hundreds of water molecules. A successful tetrahydrate model in a continuum solvent was developed by Nagy for the *syn-anti* transformation of the acetic acid carboxylic group [79], whereas three waters in hydrogen bonds to the solute were not enough for modeling the immediate solvation environment of the transition state for 2F-phenol [15].

(4) In general, only the first solvation shell around the polar sites can be modeled. Moreover, even in these cases, the explicit-solvent/continuum interface suffers from neglecting the consideration of the solvent-solvent hydrogen bonds. For methanol or acetonitrile solvents, the problem is not dramatic since the polar site of the solvent molecules should point toward the solute while the methyl group is located mainly on the outer surface of the supermolecule. Then the first-sphere solvent molecules can create a non-polar surface toward its continuum representation. This is surely not the case for explicit water molecules and is likewise questionable for a solvent like acetic acid with two stericly separated polar sites.

(5) Geometry optimization for a supermolecule leads to an overly ordered structure, which is not maintained due to thermal disordering in a real solution.

(6) If one wants to study the structure of a dilute or moderately concentrated (1 molar) solution as well as solute dimerization, boxes of a large number of explicit solvent molecules should be considered. These studies typically then require Monte Carlo (MC) [80] or molecular dynamics (MD) [81] simulations.

During MC calculations, the solution model is a large solvent box with hundreds of solvent molecules and one or a few solute molecules embedded in the solvent. Atoms are represented by point-like centers characterized with atomic charges and assigned van der Waals parameters. The interaction energy of the atoms in different molecules is calculated by pair potentials and the total energy is the sum of these pair-interaction energies. Macroscopic thermodynamic quantities are estimated by averaging the individual values calculated for a large number of consecutive configurations. A configuration means a specific geometric arrangement of the elements in the solution box. The method is a probability method, meaning that a new configuration with modified geometric arrangement of the elements is considered for the above averaging upon the probability of the acceptance of the total energy change. If the new configuration is rejected, the old one is considered one more time in the averaging process. The most frequently applied sampling procedures are the Metropolis procedure [82] or some suitable procedure [83,84], which can accelerate the convergence of the calculated averages for thermodynamic quantities or help to more quickly reach an equilibrium solution structure by applying a biased energy calculation and probability for the acceptance of a new configuration. The goal is to generate a series of configurations corresponding to the Boltzmann distribution. For constant temperature simulations, the temperature is a parameter of the expression determining the acceptance probability. After having generated the required set of configurations, the average energy, enthalpy, volume, *etc.*, can be calculated as an arithmetic mean of the individual values obtained with each configuration. If a biasing sampling was used, the probability of the acceptance has to be corrected before calculating averages.

The MD simulation is a deterministic process. The solution box is established as described above, but the atomic masses are also considered. A force field is used for calculating the total energy of the system with a given geometric arrangement (with Cartesian coordinates for each atom) at a reference time “t”. The force field contains terms accounting for the energy contributions by atoms bound along a 1–2–3–4 path, as well as for interactions of more remote atoms within the molecule and with all atoms in other molecules. The system generally is not in energy minimum, thus there are forces acting on the atoms. Using the gradient of the total energy, the forces acting on each atom can be determined. Applying Newton’s law, the position of the atoms at *t* + Δ*t* can be calculated by means of the determined instantaneous velocities. Δ*t* must be small, generally being chosen between 0.5 and 2 fs. In the latter case, the *X*–H distances are kept at a constant value. The temperature is related to the sum of the atomic kinetic energies. The simplest way to keep the temperature at a constant value is by scaling the determined atomic velocities or by coupling a thermostat to the system. Letting the simulation run long enough, sometimes for tens of nanoseconds, the average of the thermodynamic quantities can be obtained for a simulation period, where some structural characteristics, e.g., the solution density, have reached an equilibrium value already. By examining the trajectories calculated for geometric parameters of the solute, structural changes can be followed.

Using intermolecular pair-potentials such as OPLS-AA [85,86], Amber [87] or CHARMM22 [88], the largest problem is the development of the relevant atomic charges for the molecule. For example, Amber was originally parameterized for biopolymers and DNA, and no special charge parameters were available for, e.g., the HO–CH_*x*_–CH_*x*_–*Y* (*Y* = OH, NH_2_, NH_3_^+^, *x* = 1, 2) substructures. Furthermore, the OH and *Y* charges (and the CH_*x*_ values, as well) should be conformation dependent, since, e.g., there is an intramolecular H-bond for the tG^+^g^−^ conformer of 1,2-ethanediol, which is missing in the tTt form (Figure 2). Also, conformation dependent charges have to be used with *Y* = NH_2_, and NH_3_^+^ for the OCCN *gauche* and *trans* structures [89]. Recent developers of force fields suggest using molecular electrostatic potential (MEP) fitted charges, where the MEP should be obtained for the in-solution optimized solute. Since the solute and the continuum solvent mutually polarize each other, the MEP obtained at the end of the SCF procedure for the geometry optimization and energy minimization, reflects the electrostatic potential of a polarized solute in a polarized solvent environment. Charge fitting is a working tool, although different fitting methods (ES [90], RESP, [91], CHELPG [92]) lead to different results. Nonetheless, the problem is again of how to optimize the solute in a solvent environment. The MEP for the supermolecule is not relevant, since the solvent molecules are not so strictly bound to the solute due to thermal disorientation in a large solvent box as would follow from the structure of an optimized supermolecule. Furthermore, there is generally some charge transfer between the elements of the supermolecule. Although the programs force the charge of the total supermolecule to zero or some +/− integer for ionic solutes, the individual charges for the solute and the surrounding solvent molecules generally differ from 0, +/−1 *etc.* Thus, the MEP- fitted charges for a supermolecule should not be directly accepted for the atoms of the solute and applied in the MC of MD intermolecular pair-potentials. A possibility is that the solute geometry is accepted from the optimized supermolecule, and the MEP is fitted for the pure solute in a single-point calculation. This is, however, not a consistent procedure. The author has not found a good solution for this problem when surveying the literature.

Despite the listed potential problems, the continuum solvent approach has been one of the most frequently used theoretical methods for characterizing the geometry and the energy/free energy for dissolved molecules. Since chemical equilibria depend on relative rather than absolute free energies, the problems mentioned above may not emerge in every case so harshly, and the errors could be partially cancelled. For example, the energy minimization in the supermolecule approach leads to too tightly bound water molecules. In a model, where the thermal disordering effect is also taken into consideration (MC and MD), a more loosely bound first solvation shell is expected. Nonetheless, since relative energy data are to be compared for the supermolecules with different solute conformations, the error is probably decreased. Also a more or less cancelled error may be expected regarding the interaction energy between the explicit solvent molecules at the outer surface of the supermolecules and the continuum. In a study for a series of compounds, it is a good practice to compare the computational results with available experimental values. Unfortunately, however, good-quality experimental results generally not are available in the literature for equilibria, where a number of conformers have been detected in solution.

### 2.3. Free Energy Calculations

The focus of this subsection is the free energy calculation for explicit solvent models. The continuum-solvent calculations characterized above can provide free energies for individual solutes in any conformation, protonation state and tautomeric form. The method is not well suited, however, to the problem under investigation in the present survey pertaining to the possible disruption of intramolecular H-bonds in protic solvents. Since calculation of relative free energies is satisfactory for finding the more stable conformation, a perturbation method can be utilized in simulations where large solvent boxes including a solute and explicit solvent molecules are considered. The perturbation procedure is based on the work of Zwanzig and Jorgensen [93,94] and is widely used as the “free energy perturbation (FEP) method”.

When the FEP method is applied, the atoms of solute and solvent molecules are characterized as sets of point charges with assigned van der Waals parameters. Locations of the solute charges symbolize those for atomic nuclei in the molecule. The combined effect of the accepted net solute charges should reproduce the in-solution molecular electrostatic potential nearly within and out of the van der Waals surface. To achieve this, it is useful to obtain the values of the point charges and determine their locations for the involved conformers/tautomers of the solute by geometry optimizations followed by the fit of charges to the in-solution MEP. In the applications of FEP below, the geometry of the solute with an intramolecular H-bond is converted into another structure without this bond. The atomic charges are also converted gradually and simultaneously from the starting set to final set, characterizing the corresponding conformations. The free energy is a state function, thus even if the intermediate states do not exist physically (when, e.g., the proton annihilation/proton development path is traveled) the sum of the perturbed free energy increments are theoretically correct. The molar free energy increment in the “i”th step, ΔG_i_ is calculated as −*RT* ln[exp(−(*E*p_i_ − *E*r_i_)/*RT*)]_av_, where the average of the exponential expression has to be calculated through a long simulation. *E*r_i_ and *E*p_i_ and are the total energies of the system per mole in a given configuration with parameters applied for the reference and perturbed solutes, respectively. The FEP in this case is led through controlled conformations or states. The constancy of the charge and geometry parameters is maintained only for a perturbation step. The actual parameters for the reference and perturbed states can be determined by a linear transformation of their values between the starting and the end structures. For example, conformations with and without an intramolecular H-bond correspond to perturbation parameters λ = 0 and λ = 1, respectively. In order to keep the free energy increments below about 4 kJ/mol, Δλ may be as small as about one hundredth.

The models used for water as solvent are generally TIP3P or TIP4P [20]. Some rigid, united atom CH*_n_* (*n* = 1–3) models also are available for small organic solvents such as CCl_4_, CHCl_3_, CH_2_Cl_2_, CH_3_CN, CH_3_OH [95] when the BOSS program (Biochemical and Organic Simulation System) [96] is used for Monte Carlo simulations [97,98,99]. The solvent models were parameterized for producing good density and heat of vaporization for the neat solvent, thus the accepted model implicitly accounts for the solvent-solvent interactions, including mutual polarizations of these molecules. Recently, however, one can create solvent boxes with all-atom solvent models by using the OPLS-AA 12–6–1 force field parameters [85,86].

**Figure 4:**
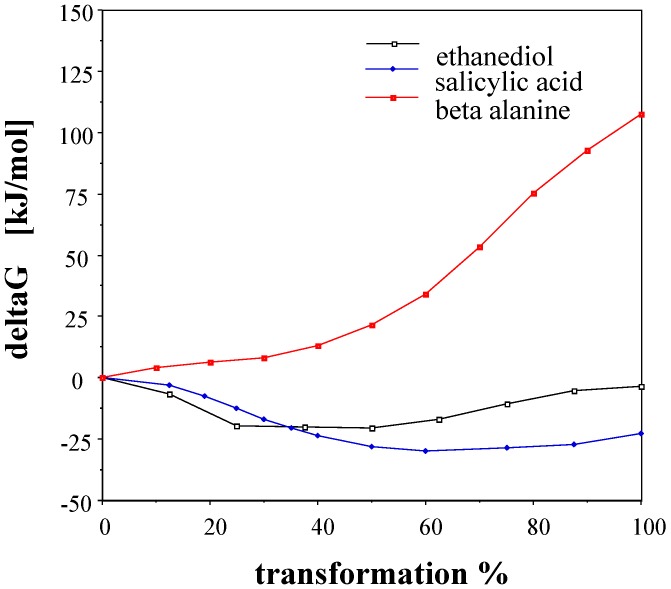
The free energy perturbation (FEP) curves for the transformations of conformers with an intramolecular H-bond to structures without H-bonds. Shown are 1,2-ethanediol (**1**) to (**2**), salicylic acid (**3**) to (**4**), and β-alanine zwitterion (**5**) to (**6**) where structure numbering is taken from Figure 2.

Figure 4 is an illustration of the course of three FEP curves for conformational changes of the same molecules schematically compared in Figure 2. It is clear that the FEP, provided as the percent transformation, is not necessarily monotonic. For the two neutral molecules the most favorable hydration can be expected at about 20% and 60% transformations between the starting and finishing local energy minimum structures. For the *gauche* zwitterionic β-alanine, the ionic sites are less open for hydration and the solvation free energy the less favorable (**5** is the *trans*, **6** is the *gauche* conformation).

In MD simulations, the solute geometry can change under the forces acting on each solute atom. Determination of bond stretching, bond bending and torsion parameters for every special molecule with possible intramolecular H-bond(s) is a very time consuming procedure. On the other hand, application of some average reference geometric parameters, stretching, bending force constants, and acceptance of average torsion potentials for these rather special molecules may lead to unreliably distorted structures in a flexible solute model. Thus use of the rigid optimized geometry could be favored from energy point of view and the computer time must be shorter if the energies of the solution configurations are to be averaged for a model with all rigid elements as compared with a slower convergence in case of a flexible solute. This option is not available for MD simulations, where individual molecular mechanics parameterization is desirable for high quality simulations. In some applications of the software, the lengths of the σ-bonds with hydrogen can be kept at fixed values although the problem of the critical torsion flexibility still remains.

A further problem related to the use of the flexible solute model is that the MEP-fitted-charges characterize only the given optimized geometry. If the solute leaves this structure even temporarily, the charges are not relevant for the new geometry. Common in most MD and MC programs used in the past twenty years, however, is that the atomic point charges do not change through the simulation. In MD simulations with flexible solute geometry, the atomic charges are kept constant even if the structure changes among quite different conformational states.

This problem may be overcome by the use of the fluctuating charge (FC) model [100,101]. This approach introduces a polarizable force field, where the atomic point charges are allowed to fluctuate in response to the environment. Accordingly, this approach can account for the conformation dependence of the charges through the calculation of the solute-solvent interaction energies relevant to the actual solute geometry. The computation time increases by only about 10%, thus the method is applicable for simulations of large systems [102,103]. Nonetheless, MD/FEP calculation using the FC model has not been found even for small molecules in the surveyed literature.

The present review concentrates on results obtained with the described methods, although more recent simulation programs allow consideration of polarization charges on the solute and induced dipoles on the solvent [104]. The QM/MM (quantum mechanics/molecular mechanics) method [105,106] corresponds to the state-of-the-art level, but no article has been found where the QM/MM procedure was used for resolving the problem addressed in the title of this paper. The Car-Parinello molecular dynamics procedure (CPMD) [107] is another high-level theoretical method that could be applied as a state-of the-art procedure. The method applies pseudopotentials and the plane-wave basis set with periodic boundary conditions. The primary advantage of CPMD in comparison with *ab initio* molecular dynamics methods is that by introducing the extended variable Lagrangian formalism, CPMD can avoid the time demanding self-consistent matrix diagonalization at every step in the trajectory. Despite the attractive features of the method, the treatment of the electronic structure allows the application of CPMD only for systems that are remarkably smaller than those which can be easily considered in classical simulations. Although the possible disruption of the intramolecular H-bond in solution has been investigated only for small solutes below, the need to consider several hundred solvent molecules must have prevented applying CPMD because no such study has been found in the literature search.

### 2.4. Dimeric Solutes

The issue to be considered in this section is similar to those that have been discussed above. Solutes with two polar sites for the monomer may form one or even two hydrogen bond(s) within a dimer, which is/are intramolecular from the perspective of this species. Then the created intramolecular H-bond(s) compete(s) with the intermolecular H-bonds between the monomeric solutes in the dissociated form and the solvent molecules. Specifically, monomers with H*X*–C=O (*X* = N, O) and N–C–OH substructures would belong to this category. Formation of an intramolecular H-bond in a four-member ring with two polar functionals would lead to a strained structure. Although covalently bound four-member rings exist, a moderately strong H-bond could not maintain this relationship. B97D/aug-cc-pvtz geometry optimization [79] found geometry parameters for the gas-phase acetic acid carboxylic group very close to experimental values [28]. Accordingly, the H…O= distance is 230 pm and the O–H…O= angle is 75.5°. This bond angle is quite far from the favorable O–H…O angle of about 170° generally found to be favorable intermolecular H-bond. Thus the OCOH moiety should not be considered to be a ring, and the better way to stabilize the H*X*–C=O or N–C–OH substructure is the formation of a dimer.

The gas-phase structure of formic acid was determined from the microwave spectrum by Lerner *et al.* [108]. The main geometric parameters were obtained by their fit to the rotational constants. However, formic acid assumes mostly a dimeric structure (FAD) in the gas phase. In a theoretical study, Turi [109] identified seven stable dimeric structures on the potential energy hypersurface. The doubly-hydrogen-bonded isomer of C_2*h*_ symmetry was found to be the most stable arrangement. Experiments confirmed this theoretical prediction. A comprehensive discussion of the formic acid dimer related issues, the paradigm of symmetric hydrogen bonding, and a collection of former experimental papers were provided by Zielke and Suhm [110]. Rotationally resolved spectra were recorded under supersonic jet conditions for the FAD by Matylitsky *et al.* [111]. With the assumption of unperturbed monomers, a center-of-mass distance of *R* = 299.0 ± 0.1 pm for the monomers within the dimer was calculated from the spectroscopic results. A recent update of the experimental results on FAD augmented with theoretical calculations up to the MP2/aug-cc-pvtz level was provided by Balabin [112].

**Figure 5:**
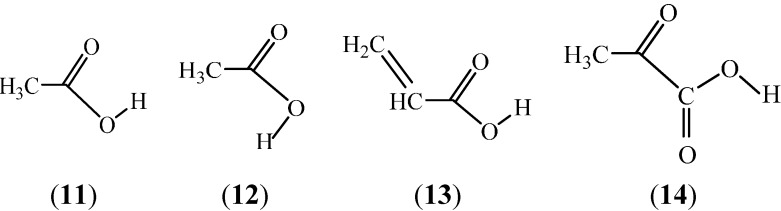
Structures of the *syn* (**11**) and *anti* (**12**) acetic acid, the *s-cis* propenic acid (**13**) and the *s-trans* pyruvic acid (**14**). The carboxylic group is *syn* for the latter two.

Geometric parameters for monomeric and dimeric acetic and propionic acids in the gas phase were experimentally determined by Derissen [28,113]. Structures of the short-chain carboxylic acids to be discussed below are shown in Figure 5.

Simple aliphatic carboxylic acids assume predominantly doubly H-bonded, symmetrical dimeric forms (52%–87%) in the gas phase. The intermolecular H-bonds for acetic acid–acetic acid methyl ester complexes were studied by Emmeluth and Suhm [114] by FT-IR (Fourier transform infrared) spectroscopy for the mixed clusters in supersonic jet expansions. The methyl ester has two hydrogen-bond acceptor sites without having a strong donor site. The structural issues for the closest analogues of the acetic acid dimer and the acid-ester complexes were discussed on the basis of the recorded spectra and theoretical calculations at the MP2/6-311++G** and B3LYP/6-31G+G* levels. An interesting question is then: Will the dimeric forms be maintained in aqueous solution, or will the stabilizing “intramolecular” hydrogen bonds become disrupted so that the monomers can form intermolecular H-bonds with the water molecules.

Another important problem is the protonation state of the elements in the complexes of short-chain acids and bases. Such hydrogen-bonded complexes are stably formed from a neutral acid and a neutral amine in the gas phase, although acid—Guanidine complexes in the form of a hydrogen-bonded ion-pair are also stable in the gas phase [115]. In aqueous solution, the acid-amine ion-pair complexes are more stable, whereas the hydrogen-bonded complexes of the neutral elements are more stable in a low-polarity environment [116]. Such studies are important for the exploration of the ligand-receptor interactions in the binding cavity of the muscarinic acetylcholine receptor. At physiological pH of 7.4, a ligand with an amine function must be mainly protonated in the aqueous environment characteristic for the extracellular side of a transmembrane protein. When the ligand penetrates into the protein, most of the surrounding water molecules must be left behind. In the binding cavity of the receptor, at about 1100 pm from the surface, the ligand interacts with an aspartic acid side chain. According to a general consensus, the side chain is originally deprotonated. Thus a theoretical study of the protonation states for the partners under the modeling condition is justified [116].

The studies for dimer/complex formations in solution start with the geometry optimization of the associated species in the selected solvent. Using the continuum solvent approach, the poor handling of the solute-solvent H-bonds is less dramatic for the dimers of aliphatic acids. The main H-bond forming sites are involved in bonds to the partner, thus a less polar molecular surface of the dimer is seen by the solvent within the cavity. An acetic acid—Alkyl amine (mainly trimethyl amine) complex exhibits even larger non-polar molecular surface. The polar sites interact with each other in the depth of the complex, irrespective of whether the H-bond formed between the constituents is neutral or ionic.

The possible dimerization/complex formation in solution can be followed by calculating the potential of mean force (pmf) curve. This curve reflects the change of the solution free energy as a function of the solutes’ separation, taking the free energy of the solution with largely separated solutes as the reference state. If the pmf is calculated by the FEP procedure, the “*R*” separation of two reference atoms of the solutes should change only slightly by, e.g., 20 pm, and the free energy at separations *R* ± 10 pm could be calculated as a perturbation. Local minima of a pmf indicate stably associated forms, local maxima indicate barrier tops for association. The pmf may have more than one minimum site, also could decrease monotonically from the large-solute-separation reference state until reaching the minimum. The latter shape of the pmf indicates solute association without activation free energy. At small solute separations generally below 350 pm, the curves steeply ascend due to the quickly increasing van der Waals repulsions.

For large, more than 500–600 pm separation of the reference atoms, acceptance of the monomeric geometry and the related net atomic charges is reasonable. When the formation of the intermolecular H-bond(s) may start between the solutes, the geometry and the charge parameters should be gradually transformed into the values that were obtained for the optimized dimer. The related charges were fitted to the corresponding in-solution MEP [79,117].

## 3. Conformational Equilibria

This section will be divided in subsections having the word “rings” in their headers. This ring refers to the H–*X*–C*_n_*–*Y* substructure moiety forming the intramolecular *X*–H…*Y* H-bond. The symbol *Y* stands either for a H-bond acceptor atom or an aromatic ring.

### 3.1. 3-Member and 4-Member Rings

Although *X*–H…*Y* interactions must be present in these “rings”, they cannot be considered as real, intramolecularly H-bonded structures. There are very few examples that may belong to this type of 3-member ring systems. DePrince and Mazziotti [40] pointed out that the nitrone structure in the form of CH_2_=NHO possesses a semipolar N^+^–O^−^ bond and a N^+^–H…O^−^ “hydrogen bond”. This arrangement corresponds overall to a 3-member ring. The structure is a local energy minimum on the potential energy surface when the tautomeric transformation from nitroso methane (CH_3_NO) to formaldoxime (CH_2_=N–O–H) is studied. The N–O–H moiety does not form a 3-member ring for the latter. The nitrone structure is more stable than the nitroso methane form in the gas phase by about 6 kJ/mol at the CCSD(T)/CBS level [40] and by 16–17 kJ/mol at the B3LYP/6-311++G** level [118]. The authors of the latter paper studied solvent effects on the tautomeric process. They confirmed that the formaldoxime form is the most stable structure in solution, but no solvent effect results were provided for the nitrone tautomer.

Alkorta and Elguero [119] studied the 1,2–proton shifts in the gas phase for 3- to 7-member, unsaturated rings with a HN–N moiety within. Through the tautomeric process they found 3-member-ring transitions states (TS) in the form of N…H…N, where the structures could formally be considered as intramolecularly H-bonded species. The calculated zero-point relative enthalpies scatter between 100 and more than 400 kJ/mol for the studied systems. The problem of forming 3-member N…H…N TS’s may also emerge for the tautomerization of 1,2,3 triazoles, tetrazoles, and for substituted 1,2,4 triazoles.

Pyrazole is a representative of the above series that has a 5-member aromatic ring. Alkorta and Elguero paid special interest to this molecule since it is an important small heterocycle in synthetic chemistry. They found that the shifting proton in the transition state stays out of the plane of the heavy atoms, whereas it is in the plane in the energy-minimum ground state. The relative TS energy and zero-point enthalpy are 214 and 198 kJ/mol, respectively, as calculated at the B3LYP/6-31G* level. However, is this intramolecular route with high activation energy is necessary for the 1,2 proton shift?

Rice *et al.*, [120] recently published a paper indicating that the pyrazole dimer was observed in a free jet expansion. Its IR-active N–H stretching frequency was red-shifted by 269 cm^−1^ relative to the monomer. The symmetry was assigned as C_2*h*_, which involves a coplanar system having two intermolecular H-bonds in a six-member ring. The 1,2-proton shift in pyrazole would result in an undistinguishable new structure. However, if there is a ring substituent in position 3, there are two, chemically different tautomers. For their equilibration, the indicated dimer structure is a convenient route through a double proton relay. The authors raised the possibility that even larger, e.g., C_3*h*_ trimers could also be formed, which are also convenient structures for proton jumps to a neighbor and accepting a proton from the other neighbor in the trimer. Unfortunately, no reference has been found in the literature that such dimers, trimers are stable in aqueous solution.

The above intermolecular proton repositioning must be a fundamental equilibration route for a number of systems when the intramolecular route requires too high activation energy. Tsuchida and Yamabe (TY) [121] proposed a tautomerization pathway between hydroxypyridines and pyridones, where one of the described paths could be applied for any system in aqueous/alcohol solutions. A simple way for tautomerization is the double proton-relay through a dimeric form. For H–*X*–(C)*_n_*–Y monomeric substructures (*X*, *Y* electronegative atoms), six-member dimeric rings could be formed with *n* = 0 (pyrazole above), eight-member rings with *n* = 1 (2-OH pyridine), *etc.* Formation and stable maintenance of such rings could be favorable in non-polar solvents, and a tautomeric process could conveniently proceed. The shown example was the 2-OH pyridine to 2-pyridone tautomerization. For the realization of the indicated reaction, the only pre-requisite is the stable maintenance of the properly oriented dimeric form.

The tautomerization is also possible through the double-proton-relay mechanism in protic solvents without, however, forming a dimer. Upon the basic idea of the TY mechanism (see above), a protic solvent having both proton donor and acceptor sites could catalyze the indicated process. In this case, a proton from the solute’s H-*X* site jumps over to the acceptor site of the closest protic solvent molecule, and a proton returns from the solvent to the solute’s acceptor site. This reaction mechanism need not be confined to the involvement of a single solvent molecule. Since protic solvents generally form a H-bonded network, a series of the proton jumps along the several-element solvent bridge will carry the extra proton to the proper site of the solute, even when the two sites of the solute are far away from each other.

The mechanism could work for the neutral form/zwitterionic equilibration of the 3- and 4-pyridine carboxylic acids [122], aminophenols [123] and for any saturated amino acid in a protic solution. Sometimes the conformational change by rotation about the (H)*X*–C bond may lead to the disruption of the intramolecular H-bond for small organic solutes in water/alcohol, but the possibility of the solvent catalyzed disruption of this bond through the corresponding TY mechanism was also considered as a competing reaction path for, e.g., 2-F– and 2-Cl ethanol [15].

Overall, the tautomerization through dimerization or by solvent catalysis could be a likely, low-activation-energy mechanism in the case of a *X*–*X*–H substructure, as for 3-substituted pyrazoles, and must be very important in the case of a H–*X*–C–*Y* tautomerization to *X*–C–*Y*–H, as well. The chance for an intramolecular proton transfer may start for structures where the *X*H…*Y* moiety is involved in a ring with at least five members and the chain is flexible for forming a favorably short H…*Y* separation. A typical example is the zwitterion formation for α-amino acids (see Subsection 3.2.2).

The amide and carboxylic groups are two well-known representatives of the H–*X*–C–*Y* substructures. In proteins, the H and O atoms of the H–N–C=O peptide bond are in *trans* position and there is no possibility to form an intramolcular H-bond, not even upon considering the distance criterion. However, one of the protons points toward the carbonyl oxygen in free amides with a H_2_N–C=O substructure allowing for the formation of the intermittently stable HN=C–OH tautomeric species.

The hydroxy hydrogen in *syn* carboxylic acids (Figure 5) is at a distance of about 230 pm from the carbonyl oxygen, and the O–H…O angle could be as small as about 76° (see above [79]). The *syn* –COOH group is coplanar for a monomer. Without a deeper analysis of the molecular orbitals, a simple explanation for the *syn* –COOH preference may be that the electrons of one of the carbonyl’s lone pairs face the carboxylic hydrogen in the *syn* conformation, whereas this lone-pair would see the lone pairs of the hydroxy oxygen in the *anti* form. Thus, a conclusion here is that the dominant conformation is basically directed by electrostatic forces. Nonetheless, the solvent effects are more favorable for the *anti* rather than the *syn* form in aqueous solution [79,124], so an observable *anti* conformation of the acetic acid in aqueous solution is likely.

The tetrahydrate model of acetic acid placed in a cavity in a continuum water solvent [79] facilitates the *syn* to *anti* conformational change not by rotation about the C–O(H) bond, but by a double proton relay involving the TY mechanism. However, it was pointed out in the same study that the *syn* dimeric form of acetic acid is present in a large fraction in molar aqueous solution, thus the *syn* to *anti* transformation is feasible only for the free acetic acid that is expected to predominate in very dilute solutions.

According to the literature search, the acetic acid dimer has been the subject of the most studies dealing with a dimeric system in the liquid phase. This compound assumes about an 87% dimeric form in the gas phase [28] and forms different, mainly cyclic and linear intermolecular H-bonds in neat liquid [125]. The in-solution association depends on the pH of the solution. The p*K*_a_ of this molecule is 4.76. A simple calculation concludes that if one mole of this acid dissolves in pure water less than 1% of the solutes dissociate, and the pH of the solution decreases to about 2.4. For a 0.1 molar aqueous solution, the degree of the dissociation is slightly larger than 1% and the pH is about 2.8. Thus, under such conditions, consideration of the neutral form as the prevailing protonation state is justified. Acetic acid is, however, almost fully dissociated at pH = 7.4 under physiological conditions.

The acetic acid dimer has been investigated theoretically in several different studies during the past decade. Yamabe and Tsuchida [126] studied the water-catalyzed process: Acetic acid dimer → monomer → dissociation (ionization). When a water cluster attacks the dimer, the monomeric and later the ionized form come into existence through the formation of several unstable and stable intermediate structures. This mechanism is important to understand the formation of the hydrated acetate ion, even though the experimental p*K*_a_ suggests that only a small fraction exists in the ionized form in pure water.

For obtaining the pmf via the MC/FEP method, Nagy [79] considered two charge sets. The first set was derived for the monomeric acetic acid optimized at the IEF-PCM/B97D/aug-cc-pvtz level in a continuum water solvent and the charges were fitted to the in-solution MEP by the CHELPG process. In this first approximation, the charge set was applied along the whole considered R(C…C) separation range of 314–1184 pm (C is the carboxylic carbon). The intermolecular H-bonds start forming at *R*(C…C) 484 pm and two strong intermolecular bonds are expected to come into existence at about 386 pm, which is the optimized C…C distance for the dimer at the IEF-PCM/B97D/aug-cc-pvtz level. The calculated atomic charges were remarkably different in the dimer compared with the monomer. The dimer charges were then applied in the C…C range of 484–384 pm in the second approximation. The charges and the geometries were gradually transformed from the monomer to dimer values in this range. As a result, the pmf showed a minimum deeper by 21 kJ/mol than when monomer charges were only used. Following the method of Ciccotti [127], upon the integration of the *R*^2^ exp(−*G*(*R*)/*RT*) curve (*R* is the C…C separation, *G*(*R*) is the free energy of the system with reference *G* = 0 at 1184 and 261 pm for the molar and 0.2 molar solutions, respectively), the predicted association degree was 38%–45% and 9%–10% at the two concentrations when the first charge set was applied. In contrast, almost 100% association was predicted for a molar aqueous acetic acid solution by the second approach. However, the deepening of the pmf minimum value is overestimated in the second approach because the internal free energies of the solute partners have to increase at that separation. Indeed, under the conditions where a monomer has a geometry and atomic charges equal to those which were derived for the elements of the optimized dimer, the monomer’s internal free energy must be, by definition, higher than that for the optimized, separate monomer in water. Thus a correction (not carried out in [78]) must diminish the difference in the minimum *G* values upon the two approaches.

There are four pmfs presented by Chen *et al.*, [128] for formic, acetic, propionic and butyric acids in aqueous solution. The calculations utilized the CHARMM22 all-atom force field. The *G* = 0 reference state was taken at *R*(C…C) 1100–1200 pm. All pmfs show a clear minimum at carboxylic C…C separation of 400–500 pm. Whereas the valleys around the well-defined minima are narrow for HCOOH and CH_3_COOH, and the pmfs show a small barrier with slightly positive relative *G* for desolvation, the free energy is negative for butyric acid up to *R* about 1100 pm. More importantly, the butyric acid pmf runs remarkably below the acetic acid pmf in the *R* = 600–800 pm range. Thus the pmfs are similar in the intramolecular H-bond formation range but the favorable interactions of the non-polar, aliphatic chains at *R* > 600 pm help increase the readiness for solute association even with substantially separated carboxylic groups. The derivable calculated association degree for CH_3_COOH is 37% in a molar solution, close to the results of Nagy above when the first charge set was used. Both theoretical predictions overestimate, however, the upper limit experimental value of 14%, when using p*K*_D_ from Table I in [126]. In fact, all theoretically predicted p*K*_D_ values are overestimated but the linear correlation with the upper limit experimental values is good, providing 0.97 for the square of the correlation coefficient.

Chocholoušová *et al.*, [129] concluded that whereas acetic acid takes a doubly H-bonded cyclic dimeric form in the gas phase, the monomeric form prevails when it is dissolved in aqueous solution as found from molecular dynamics simulations. The association state is still a doubly H-bonded cyclic dimeric form in chloroform. The presumably Amber NpT molecular dynamic simulations were performed in the range of 0.3 to 10 molar concentration in aqueous solution. The applied RESP charges [90] were fitted to the HF/6-31G* wave function. These charges must differ remarkably from those derived by Nagy above, and probably differ also from those utilized by Chen *et al.* above. The different charge sets may lead to very different conclusions.

Recently Pašalić *et al.*, [130] carried out a DFTB [131] molecular dynamics simulation for the acetic acid dimer within a box of 200 water molecules (about 0.5 molar). In such calculations it is not necessary to predefine a set of net atomic charges, because the interactions between the solute and solvent molecules are recalculated quantum mechanically in every step. The calculations starting from different dimer geometries were relatively short, lasting only for 300 ps. Nonetheless, the authors found that the elements of all acetic acid dimers started to dissociate after about 50 ps. Although a DFT-based molecular dynamics is a large progress compared with MD simulations utilizing empirical force fields with set atomic charges, these calculations predict energies in the NVT ensemble, and according to Elstner *et al.*, [131] “… in order to compare to experimental situations, free energies rather than potential energies have to be calculated. This would involve molecular dynamic (MD) simulations over rather long time scales, ranging from several hundreds of picoseconds up to milliseconds.” Thus the simulations conducted by Pašalić *et al.*, [130] must be at the lower limit at best.

On the experimental side, dimerization constants for acids have been determined by several groups, as shown in [126]. D’Amico *et al.*, [132] performed inelastic UV scattering experiments on aqueous acetic acid solutions at different temperatures and solute concentrations. The author found a crossover temperature, *T*_c_ ≈ 325 ± 10 K above which “the energy of hydrogen bonds responsible for water-acetic acid and acetic acid-acetic acid interactions is strongly reduced. This leads to a reduction in the average number of water molecule interacting with acetic acid, as well as to a lower number of acetic acid clusters.” Thus at about 300 K, where the MC and MD simulations were conducted, existence of acetic acid clusters has been concluded by D’Amico *et al.*

In summary, experimental data predict association for acetic acid in dilute aqueous solution, although the degree of association may be only 14% or less. This result has not been successfully reproduced theoretically. Different approaches lead to different results including both contradicting and overestimating of the association.

Close *X*…H distances can appear in the 2-OH and 2-SH furan and thiophene in the *X*–C–*Y*–H *cis* conformation and also for 3-OH pyrazole, 3-OH and 5-OH isoxazole. These structures are not considered to have an intramolecular H-bond within their four-member rings, although the conformational preference compared with the *X*–C–*Y*–H *trans* form is interesting and could be affected by the solvent. Old HF/STO-3G calculations by Radom *et al.*, predict OH *cis* and *trans* conformations for furan and thiophene rings, respectively, in the gas phase [133]. No recent solvent-effect calculations have been found.

For 3-OH and 5-OH isoxazoles, a number of theoretical calculations have been carried out in aqueous solution without uniform conclusions [134,135,136]. Different solvation methods and internal energy calculations indicate that the structural problem is subtle. The systems are subject to oxo-hydroxy tautomeric transformations, and the possible intramolecular H-bond issue was not of interest in the studies. In contrast, the conformational problem was investigated for the hydroxy tautomer. For 3-OH isoxazole [134], the NCOH *cis* form is more stable than the *trans* in the gas phase by 18.0 kJ/mol at the MP4/6-31G**//3-21G level. By combination of the relative gas-phase energy with the relative solvation free energy from an MD/FEP calculation in aqueous solution, the *cis* form still remains the more stable conformer by 10.9 kJ/mol. In the same paper, the *cis* O–C–O–H conformation of 5-OH isoxazole was found to be more stable than the *trans* form by 8.4 kJ/mol in the gas phase and by 2.1 kJ/mol in solution. Gould and Hillier [135] also found the *cis* O–C–O–H form to be more stable than the *trans* structure by 1.3 kJ/mol in aqueous solution. Thus the solvation by water favors the *trans* conformer, but the *cis* structure is still maintained for the hydroxy forms of substituted isoxazoles.

Cramer and Truhlar [136] studied the conformational/tautomeric problem for the 5-OH isoxazole. The free energy difference in the gas phase at *T* = 298 K is less than 1 kJ/mol in favor of the *cis* form as determined from high-level ab intio calculations. The aqueous solvation favors the *trans* form by about 1.7 kJ/mol, thus the *trans* O–C–O–H conformation was predicted by these authors as the more preferable conformation by up to 1.7 kJ/mol in aqueous solution, depending on the applied models. The *trans* form also remains the more stable conformer for the 3-methyl and 3,4-dimethyl derivatives by up to 3 kJ/mol.

### 3.2. 5-Member Rings

This is a large family of small organic molecules where an intramolecular H-bond is supposed to exist, although AIM analyses may sometimes point out its absence. Nonetheless, close H…*Y* distances have been calculated and experimentally found in the gas phase for a number of such structures. A schematic structure for 2-Cl ethanol, as a prototype with a five-member ring is shown in Figure 1. Schemes for a *gauche* and the all-*trans* conformations for 1,2-ethanediol, as well as a similar pair of the zwitterionic β alanine are provided in Figure 2. *Gauche* conformers for 2-aminoethanol and 2-NO_2_-ethanol (the latter forms a six-member ring in the internally H-bonded structure) with and without intramolecular H-bonds are shown Figure 3.

#### 3.2.1. 1,2-Disubstituted Ethanes and Derivatives

1,2-Dihydroxy ethane (1,2-ethanediol). This molecule has been studied experimentally in the gas phase, organic solvents, in water, and theoretically both in the gas phase and in aqueous solution. A detailed summary of the conformational issue can be found in [22]. A concise summary is provided below.

On the basis of gas-phase electron diffraction results of Bastiensen [137] and the Hedberg group [30], microwave spectroscopy results of Caminati and Corbelli [138] and IR studies by Frei *et al.*, [139] and Takeuchi and Tasumi [140], the 1,2-dihydroxy ethane (1,2-ethanediol, ethylene glycol) is a mixture of two main conformers, tG^+^g^−^ and g^+^G^+^g^−^ in the gas phase with intramolecular H-bonds. Capital G refers to the *gauche* arrangement of the OCCO moiety, t and g^+^ refer to the free *trans* and *gauche* hydrogen in the HOCC moiety, respectively, whereas g^−^ stands for the internally bound other hydrogen atom, which is in an electrostatically favored position. However, no (3, −1) BCP was found for this arrangement [6,7]. Quantum chemical studies by Nagy *et al.*, [42,43] and Cramer and Truhlar [44] also found these two conformers as the most stable ones in the gas phase. Cramer and Truhlar obtained their relative energies at the high (MP2/cc-pvtz + CCSD(T)/cc-pvdz-MP2/cc-pvdz) theoretical level. The “+” and “–” superscripts follow the code in this article.

Krueger and Mattee [141] interpreted the structural results on the basis of temperature dependence of the fundamental OH stretching bands in carbon tetrachloride so that the OCCO *gauche*, *G* conformation is overwhelming in organic solvent with one or two intramolecular hydrogen bonds. Pachler and Wessels [142] also found the G arrangement the predominate for 1,2-dihydroxy ethane in solution with up to 20% OCCO *trans* fraction in different organic solvents with low dielectric constants and 12% *trans* conformer in *D*_2_O-solution.

Nagy *et al.*, [42,43] predicted a 99.5% G fraction on the basis of *ab initio* MP2/6-31G* + MC simulations, considering, however, only five G and one T conformers. There are altogether ten stable conformations for this molecule: six G and four T. Consideration of the missing 3T and 1G conformations would likely increase the overall T fraction. Such MC free energy perturbation calculations were, however, extremely time consuming in the first half of the 1990’s. Overall, the studies predicted about 67% and 33% conformers without and with an intramolecular H-bond in solution, respectively, thus the short H…*Y* distance in the gas phase was mostly eliminated upon interactions with water molecules.

Cramer and Truhlar [44] studied all ten conformers using a continuum solvent approach. They successfully predicted about 12% for the OCCO T conformation, and predicted 36%–54% tG^+^g^−^ conformer in the equilibrium solution as compared with 56% in the gas phase. Thus their results correspond to a major in-solution conformation with a favorably close O–H…O arrangement. Altogether 73%–84% of the conformers were found with arrangements favorable for an intramolecular H-bond. The deviation between the structure-predictions in this study and those above indicates the possible underestimation of the significance of the solute-solvent intermolecular H-bonds by continuum solvent methods. Hooft *et al.*, [143] studied the conformational problem by means of explicit–solvent molecular dynamics simulations and utilized the GROMOS force field. They predicted a G:T = 67:33 ratio and altogether 31% for conformers with a possible intramolecular H-bond.

The three investigations result in rather diverse results. Nonetheless, there is a unanimous conclusion that the OCCO moiety takes predominantly the G arrangement in solution, similar to that in the gas phase. Cramer and Truhlar found about 12% T conformation in good agreement with the experiment. The T fraction was under and overestimated in the other two investigations. The three studies differ mainly in predicting the fractions of conformers regarding the O–H…O substructure. Nagy *et al.*, and Hooft *et al.*, predicted the majority of structures with fairly large H…*Y* separation, Cramer and Truhlar found the opposite.

In a very thorough analysis by Petterson *et al.* [144], the G *vs.* T conformation problem was interpreted from a new perspective. After reviewing many former experimental studies, the authors discussed their own NMR results for the solution of 1,2-ethanediol in DMSO-d_6_ and CDCl_3_. They concluded that the OCCO *gauche* conformers (G) are prevailing, but their fraction strongly depends on the in-solution OCCO torsion angle. Nevertheless, the reason for the G preference for 1,2-ethanediol and 2-fluoroethanol (see below) is the well known “*gauche* effect” noticed for several 1,2-disusbstituted ethane derivatives, and the possible intermolecular H-bond is not a decisive factor to stabilize the G form. Accordingly, the intramolecular H-bond may or may not be maintained in aqueous solution for a G conformer, whereas the OCCO *gauche* arrangement remains predominant.

Gubskaya and Kusalik [145] performed MD simulations with 1,2-ethanediol molar fractions of *X* = 0.03, 0.1, 0.3, and 0.8 in ethanediol:water mixtures. The molar fraction in the experiment by Pachler and Wessels [142] was about 0.08. Gubskaya and Kusalik found sensitive dependence of the conformer population on the concentration of the system. Comparison is reasonable only up to *X* = 0.3, since *X* = 0.8 corresponds to a solution where the organic portion should be considered as the solvent.

The population of the OCCO *trans* (T) conformation was calculated at 56% with *X* = 0.03, but no T was found at *X* = 0.1 Interpolating the T population to the experimental *X* = 0.08 composition, where the finding was 12% T, the agreement is quite good. The total *gauche* fraction in dilute solution has contributions from two different OCCO *gauche* structures. Although the torsion angles are ± 62.5° for G' and G, they must not be optical antipodes since their populations are 8.7% and 35.3%, respectively. This author could not determine the torsion angles for the hydroxy hydrogens, so it was not clear whether any of the G' and G conformers would refer to a tG^+^g^+^ or a tG^+^t conformation without an intramolecular hydrogen. Nonetheless, one may conclude that 44% of the solutes maintain some *gauche* conformation allowing for the existence of structures with an intramolecular H-bond in aqueous solution. Since the calculated compositions are highly concentration dependent, it is difficult to compare these results with the three others above, where the simulation conditions modeled an infinitely dilute solution.

1,2-Ethanediol is a substructure in sugars. The length of this review does not allow a deeper structure analysis for sugars. Nevertheless, for assignment of the OH conformations relative to the rest of these molecules by, e.g., NMR investigations [146], the above theoretical conclusions could be very helpful.

A better sugar-substructure model for tetroses, pentoses, *etc.*, would be glycerol. Glycerol (1,2,3-propanetriol) exhibits, however, a very complicated conformational equilibrium with 126 possible conformers, with or without intramolecular H-bonds in the gas phase. Bastiensen [137] studied the structure by electron diffraction, and found the αα and αγ conformers as the major species. The symbols α, β, and γ refer to the heavy atom torsional positions, irrespective of the hydroxy-hydrogens positions (see [137,147]). He concluded that there are two intramolecular H-bonds for glycerol, which have three hydrogen-bond donor (and acceptor) OH groups. Gas-phase studies by Jeong *et al.*, [147] at the M06-2X level and by Callam *et al.*, [148] at the G2(MP2) and CBS-QB3 levels deviate slightly regarding the conformer composition at *T* = 298 K. The most populated conformer is the αγ structure followed by αα or γγ. The free energies differ by less than 1.4 kJ/mol for the two most stable species by the different methods, thus the results are fairly method and basis set dependent. In aqueous solution, the theoretically predicted fractions with the six back-bone combinations of the individual α, β, and γ torsions are in better agreement with the experimental NMR results of Van Koningsveld [149] at the SMD M06-2Z/cc-pvtz level by Jeong *et al.*, than when Callam *et al.*, (see above) applied the SM5.42//HF/6-31G* approximation. Nonetheless, both groups of authors used continuum dielectric solvent models without explicitly considering water molecules in the first solvation sphere. Accordingly, Jeong *et al.* found five conformers within a 0.7 kJ/mol range for relative free energies. For these structures two intramolecular H-bonds are maintained.

In a recent MD simulation, Egorov *et al.*, [150] studied 5.1 and 7.6 molar aqueous solutions of glycerol and neat glycerol. These solutions cannot be considered as dilute ones. Not surprisingly, the conclusion is that the typical glycerol hydrogen-bond network still exists even in the less concentrated solution (5.1 molar, of about 40 wt % glycerol). About 25% of the solutes occur in a structure similar to that in pure liquid glycerol, *ca.* 25% takes the water-solvated monomeric form, and about 50% of the glycerols form hydrogen-bonded strings. This study also cannot answer the question whether the two intramolecular H-bonds, generally assigned to the most stable gas-phase conformers, would become partially or entirely disrupted in dilute aqueous solutions. Correct characterization of the hydrogen-bond pattern for the latter system could be interesting from a medical point of view. The sugar level in blood, basically an aqueous solution, should be below about 0.005 mol/L for a healthy person. Although MD/MC simulations can be performed generally only on the 0.01–0.1 mol/L scale as a dilute solution, such studies still could provide useful information regarding the conformations of glycerol in highly dilute aqueous solutions.

1,2-Ethanediol monoethers. Cyclic ethers have been demonstrated as strong H-bond acceptors in ether…water dimers from theoretical calculations [151]. The 1,2-ethanediol vinyl-ether takes an intramolecularly H-bonded OCCO *gauche* conformation in the gas phase, as found by Marstokk and Møllendal [24]. The methyl-ether was calculated by Gil *et al.*, [152] as forming an intramolecular H-bond in the gas phase, which is maintained in CCl_4_ solution. Krueger and Mettee, who came to the conclusion on the basis of experiments conducted in dilute carbon tetrachloride solution [141].

Tafazzoli and Jalili [153] studied the conformational behavior of the 1,2-ethanediol monomethyl-ether (2-methoxyethanol) through Monte Carlo simulations in aqueous solution. They used the 12–6–1 intermolecular pair potential and the TIP4P water model in conjunction with a flexible COCCOH skeleton. The authors concluded that there are 1.73 solute-solvent H-bonds, on average, in aqueous solution with a single solute, and the water molecules favorably form H-bonds between the two oxygen atoms of the solute molecule. Using the FEP method, the potential of mean force curve was obtained in the range of 300 to 820 pm for the centers of the C–C bonds of two solutes in a water box. A double-minimum character of the pmf indicated contact and solvent separated solute associations with mainly solute…water H-bonds even in the contact-pair form.

2-Aminoethanol (ethanolamine). Penn and Curl investigated the molecular structure by microwave spectroscopy in the gas-phase [154]. The prevalent conformation is OCCN *gauche* with an O–H…N intramolecular H-bond. Because of the basic character of the amino group (p*K*_a_ = 9.5), it becomes protonated as a primary amine when dissolved in water. The protonation stops at 1%–3% for 0.1–1 molar solutions and the pH increases to 11–12. Thus the neutral form should be chosen for theoretical consideration as the major in-water component. IEF-PCM/B97D/aug-cc-pvtz studies [64] predict 89% *gauche* conformation with the intramolecular O–H…N bond, and 7% and 4% for the *gauche* and *trans* conformations, respectively, without this bond. However, when the relative solvation free energies were calculated via the MC/FEP procedure, the predicted OCCN *trans*:*gauche* (without H-bond):*gauche* (with H-bond) ratios change to about 92:6:2, indicating 98% of the conformers without an intramolecular H-bond in aqueous solution. Although in other reported cases the population orders have been generally maintained when the two solvation free energy calculations were applied, the results became essentially different in this case.

**Figure 6:**
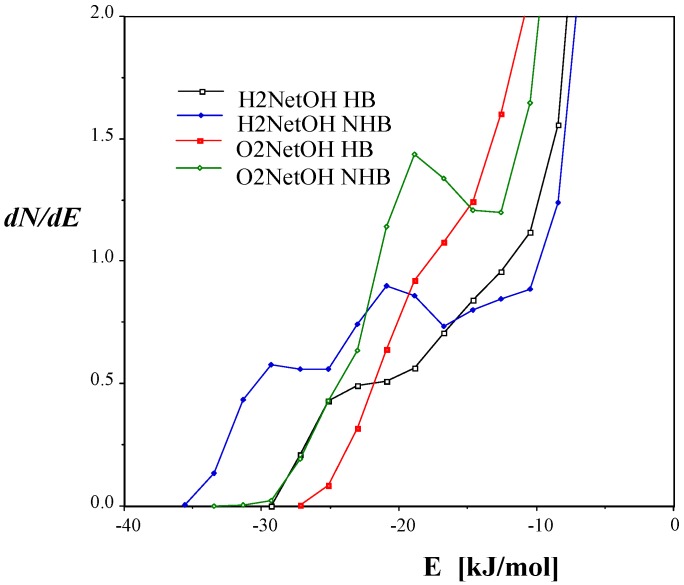
Solute-water pair-energy distribution functions for 2-aminoethanol and 2-NO_2_-ethanol with (HB) and without (NHB) an intramolecular H-bond: H_2_NetOH HB (**7**); H_2_NetOH NHB (**8**); O_2_NetOH HB (**9**); O_2_NetOH NHB (**10**). Structure numbers from Figure 3.

By considering the solute-solvent pair-energy distribution function (pedf) from MC simulations utilizing the explicit solvent model for water (Figure 6 for two 2-aminoethanol *gauche* conformers) the predicted number of H-bonds with the solvent, *n*_HB_, is considerably larger for the *gauche* conformer without than with the intramolecular H-bond. The value of *n*_HB_ in an interaction energy range can be calculated by the integration of the pedf for the range.

The HB conformer has no clearly-developed maximum-minimum shape, only an inflection point appears at *E* = −20 kJ/mol. Moreover, the pedf starts at about 7 kJ/mol less negative energy than the NHB curve, indicating that even the strongest solute-water interaction energy is remarkably weaker than that for the no-HB (NHB) *gauche* conformer. The pedf for the NHB conformer shows a double-minimum structure. The first minimum at *E* = −25 kJ/mol is the upper limit for the strong O–H…O (water) hydrogen bond formation. The second minimum appears at *E* = −17 kJ/mol. The energy range of −25 to −17 kJ covers the weaker O–H…O (water) interactions and has contributions mainly due to the H (water)…N intermolecular H-bonds. Starting at −17 kJ/mol, the two curves run fairly close to each other. This energy interaction range has been interpreted to result mainly contributions involving the N–H…O (water) weak intermolecular H-bonds, which are available for both *gauche* conformers. The pedf has not been indicated for the *trans* form, which is even more favorably hydrated. For this conformer, the polar sites are fully opened to interactions with the solvent environment.

In contrast to the above MC/FEP results, Gubskaya and Kusalik [145] found no *trans* conformer with solute molar fraction of 0.03 in MD simulations (see details above for 1,2-ethanediol). The *trans* fraction suddenly increases, however, to 66% with *X* = 0.1, and disappears again with *X* = 0.3. If these results are correct, then the composition is largely concentration dependent for the aqueous 2-aminoethanol solutions. Da Silva *et al.*, [155] found no *trans* conformer in infinitely dilute aqueous solution, where the *gauche* form maintained the intramolecular H-bond on the basis of MD simulations at 298 and 333 K. The same conclusion was drawn utilizing simulation results for the 10% (*X* = 0.1) ethanolamine solution model. López-Rendón *et al.*, [156] also predicted the internally bound *gauche* ethanolamine conformation by MD simulations at two different solute concentrations. Even the more dilute solution, *X* = 0.25 is too concentrated for reasonable comparisons with studies above.

For exploring the possibility of the formation of an intermolecularly H-bonded dimer with one or perhaps two O–H…N bonds in aqueous solution, a pmf was calculated in the C…C separation range of 324–1184 pm for two solutes [64]. Two all-*trans* 2NH_2_-ethanol molecules were allowed to approach each other in steps of 20 pm, and the deviation in free energy from the reference separation was calculated at +/− 10 pm by means of the FEP method. A shallow minimum was calculated in the 350–650 pm range. By integration of the *R*^2^exp(−*G*(*R*)/*RT* curve (*G*(*R*) = 0 at C…C = 1184 pm) only 10% of the solutes take an associated structure up to C…C = 654 pm, without exhibiting solute-solute intermolecular hydrogen bond(s) in an about 0.22 molar aqueous solution with solute molar fraction of *X* = 0.004. The MD simulations results by da Silva *et al.* [155] also suggest that ethanolamine dimer formation in aqueous solution is very limited. This is in contrast to the prediction of Haufa and Czarnecki [157] regarding intersolute H-bonds, in, however, a much denser solution with *X* = 0.6 for the solute.

In an early experiment, Omura and Shimanouchi [158] recorded the Raman spectra of 2-aminoethanol in aqueous solution at pH = 0 and 12.6. The solute is neutral at the larger pH. The authors found both OCCN *gauche* and *trans* conformers, although they could not determine the conformer ratio. NMR studies by Smith *et al.*, [159] pointed out that the OCCN G fraction stays in the range of 80%–84% when *X*, the solute’s molar fraction changes as 0.03, 0.1, and 0.3. These experimental results are in sharp contrast to the MD prediction from [144], where 66% T (and accordingly 34% G at most) was found at *X* = 0.1. Thus the experiments in [158,159] are in accord as much that there are both G and T fractions in the aqueous solution for 2-aminoethanol. The predicted G fraction is 80%–84% up to *X* = 0.3 for the solute.

The protonated structure becomes the major form of 2-aminoethanol in aqueous solution, if the pH is about 7.4 or lower. Omura and Shimanouchi [158] predicted predominantly the OCCN G conformation for the ^+^H_3_NCH_2_CH_2_OH species at pH = 0, where an intramolecular H-bond exists in the N–H^+^…O form [89]. 92% G conformation was predicted by Smith *et al.*, [159] upon NMR analysis of the G *vs.* T problem for the hydrochloride salt of 2-aminoethanol. These experiments may be considered as studies on a substructure of norepinephrine, a small molecule involved in important biological processes. The conformational equilibrium is a central problem in this species, and because of the presence of an aryl group on the C–C chain two, non-equivalent G structures exist as will be discussed in Subsection 3.3.1.

Ethylenediamine (2-aminoethylamine). There is an equilibrium of two neutral conformers separated in energy by about 1.3 kJ/mol in the gas phase [160]. Both structures possess a weak intramolecular N–H…N hydrogen bond, which becomes feasible by the *gauche* NCCN arrangement with torsion angles of 63 ± 2°. pH dependent structure analysis was performed by Omura and Shimanouchi [158], who recorded the Raman spectra in aqueous solution at pH of 3.5, 8.4, and 13.6 The molecule is a strong base, which exists in the neutral form at pH = 13.6. At pH = 8.4, most molecules take the monoprotonated form, which is the prevalent protonation state at the physiological pH of 7.4. At pH = 3.5, the dicationic form is the typical species. Despite the repulsion of the two cationic sites in the OCCN *gauche* conformation, the Raman spectra was interpreted as indicating both *gauche* and *trans* conformations for all three protonation states of ethylenediamine. No quantitative prediction of the *gauche*:*trans* ratio has been provided by the authors, however, for any protonation state of the solute.

The IEF-PCM/B97D/aug-cc-pvtz optimized (^+^H)NCCN torsion angles for the *gauche* form are 56.4° and 51.1° in water and chloroform, respectively [89]. The torsion angle is about 180° for the *trans* conformer in both solvents. The solvation favors the *trans* conformation, but the internal free energy is much more negative for the *gauche* form in both solvents. As a result, the *gauche* form with an intramolecular H-bond of the form N–H^+^…N is the prevalent conformation in both solvents by a relative free energy of at least 11 kJ/mol.

Gubskaya and Kusalik [145] found about 67% of the *trans* form with a solute molar fraction of 0.03 for the neutral conformer, which must be the dominant protonation form if the solute dissolves in pure water. A MC/FEP pmf study for the dicationic species by Boudon and Wipff [161] indicates the preference of the all-*trans* conformation without the possibility of an intramolecular H-bond in aqueous solution.

Table 1 summarizes the applied experimental methods and theoretical calculations for resolving the conformer equilibrium problem for *X*–CH_2_–CH_2_-*Y* systems (*X*, *Y* = OH, NH_2_). The table compares studies for the three 1,2-disubstituted ethane derivatives mostly referred to in this review.

**Table 1:** Comprehensive summary of the applied experimental and theoretical methods for the conformational equilibria of some 1,2-disubstituted ethane derivatives in aqueous solution.

Structures	Theor. Calc.	Gas-Phase	Aqueous Solution					
	Theor. Ref. ^a^	Exp. Ref. ^b^	*E*_int _^c^	ZPE/G_th_^d^	Cont. Solv. ^e^	MC/FEP ^f^	MD/FEP ^g^	Exp. Ref.
1,2-Ethanediol		[137,138,139,140]						[141,142,144]
	[42,43]		MP2/6-31G*	+ ^h^		OPLS		
	[44]		MP2/cc-pvtz + CCSD(T) corr.	+	SMx			
	[143]						GROMOS	
	[145]						Amber	
2-Aminoethanol neutral		[154]						[158,159]
	[64]		IEFPCM/CBS	+	IEFPCM	OPLS		
	[145]						Amber	
	[155]						Amber	
	[156]						own FF ^i^	
Protonated								[158,159]
	[89]		IEFPCM/CBS	+	IEFPCM	OPLS		
Ethylenediamine neutral		[160]						[158]
	[145]						Amber	
Monocation	[89]		IEFPCM/B97D	+	IEFPCM	OPLS		[158]
			aug-cc-pvtz					
Dication ethylenedime								
	[161]					OPLS		[158]

^a^ Theor. Ref.: Theoretical reference; ^b^ Exp. Ref.: Experimental reference; ^c^ *E*_int_: Intramolecular energy; ^d^ ZPE/G_th_: Zero point vibrational energy + thermal Gibbs correction at *T* = 298 K; ^e^ Cont. Solv.: Continuum solvent; ^f^ MC/FEP: Free energy perturbation method through Monte Carlo simulations; ^g^ MD/FEP: Free energy perturbation method through molecular dynamics simulations; ^h^ +: The “+” sign indicates that ZPE/G_th_ was calculated; and ^i^ FF for reference [156] means force field.

2-Halogen ethanol. 2-F and 2-Cl ethanol adopt the OCCX (*X* = F, Cl) *gauche* structure and develop O–H…*X* intramolecular H-bonds in the gas phase, at least on the basis of the distant criterion and according to electron diffraction and microwave spectroscopy results [29,162]. The two structures were recently studied theoretically by Nagy [15] both in the gas phase and in solution. The calculated gas-phase geometric parameters were in good agreement with the experimental ones. In chloroform and aqueous solution, the calculated OCCF gauche fraction of about 88%–98% reproduced well the experimental values of 95%–98% by Pachler and Wessels [142]. The internal H-bond remains in CCl_4_, whereas nearly equal populations were calculated for the OCCF *gauche* fractions with and without the intramolecular H-bond in aqueous solution. In the article of Petterson *et al.* [144], the authors argue in favor of the *gauche* conformation due to the generally preferable “*gauche* effect” and do not consider the possibly only very weak O–H…F bond as a structure determining factor. No experimental conformational composition has been found for 2-Cl ethanol in solution. The calculated *gauche* OCCCl fraction was about 92% in chloroform, which decreased to 51%–86% in aqueous solution depending on whether the IEF-PCM or the FEP/MC method was used. The increasing fraction for the *trans* conformation (14%–49%) and the disruption of the intramolecular H-bond must be related to the explicit consideration of the water molecules.

2,2,2-Trifluroethanol (TFE). This compound has a remarkable effect in modifying the secondary structure of proteins as a co-solvent in TFE/water mixtures utilized in NMR studies [163] or toward stabilizing intramolecular H-bonds with carbohydrates [164]. The molecule has two main conformations, with HOCC *gauche* or *trans* positions. In the *gauche* conformation, an O–H…F intramolecular H-bond is feasible, similar to that for 2F-ethanol. In an early gas electron-diffraction experiment the vague results due to technical difficulties prevented the identification of the *gauche* and/or *trans* conformation [165]. The microwave spectrum and the OH rotational dynamics for the *gauche* conformer were studied by Xu *et al.* [166]. Durig and Larsen [167] recorded the far- and mid-IR, as well as the Raman spectra for this molecule in the gas phase. By fitting a torsion potential curve for the hydroxy hydrogen rotation to the experimental data, they predicted that the *trans* conformer is higher in enthalpy than the gauche structure only by 19 cm^−1^ (0.2 kJ/mol). Senent *et al.*, [16] overviewed a number of former gas-phase experiments and found that the results uniformly assign the *gauche* conformation to the most stable species in the gas phase. Regarding a second form as a *trans* conformation, if it exists at all in the gas phase, the predicted relative energies scatter in a wide 19–1161 cm^−1^ (0.2–13.9 kJ/mol) energy range.

Neat liquid was studied by Radnai *et al.*, [168] and Bakó *at al.*, [169] by performing X-ray and neutron diffraction studies. From the latter study, the *gauche*:*trans* ratio is 60:40 in neat liquid and the TFE molecules have about 1.6 H-bonded neighbors. TFE forms both cyclic dimers and so-called gel structures with 3–4 intermolecularly H-bonded species, involving both *gauche* and *trans* TFE conformers.

Whereas experimental results are available for the gas phase molecule and for the neat liquid, no experimental or theoretical study has been found regarding the structure of TFE in aqueous solution. The closest relationship can be revealed by the paper of Senent *et al.* [16], who calculated TFE monohydrates. The zero-point enthalpy difference for the isolated *gauche* and *trans* TFE conformers is 6.8 kJ/mol at the MP2/cc-pvdz level in favor of the *gauche* form. Monohydrate relative enthalpies at 0 K were calculated with optimized geometries for the complexes. The isomeric structure, where the water molecule forms a bridge between the O–H group and one of the F atoms of the *gauche* TFE, acts as a H-bond donor to F and corresponds to a H-bond acceptor to the OH group, is the most stable arrangement. AIM analysis found two BCP’s and one RCP for this species, which is a counterpart of the 1,2-ethanediol monohydrate identified by Klein [6].

In other three monohydrates, where the water is an acceptor to the *trans* (O)H, or is a donor to the *gauche* or *trans* OH, the enthalpy is higher than the most stable one by 9.3–16.1 kJ at the indicated theoretical level. All these structures form an intermolecular H-bond as confirmed by one BCP for each of them. The *trans* monohydrates are always higher in enthalpy than the *gauche* counterparts. Since the lowest two differ by 9.3 kJ, by more than the non-hydrated *gauche* and *trans* TFE do, the calculations make a hint that the solvation in bulk water could still favor the *gauche* conformation, but the possible presence of the *trans* form may not be ruled out.

2-NO_2_ ethanol. Although the internally bound structure should form a six-member ring (Figure 3), it is more consistent to discuss this molecule next to the other 2-substituted ethanols. The NO_2_ group is a weak H-bond acceptor, still the prevailing species of 2-NO_2_ ethanol is the OCCN *gauche* arrangement. The gas-phase microwave spectrum by Marstokk and Møllendal [25] was interpreted by hypothesizing an intramolecular H-bond. Theoretical calculations found that the conformation, which allows for the formation of an O–H…O(N) H-bond in the gas phase, is maintained in chloroform and also dominates the in-aqueous solution conformer composition [64].

The water-solute pair-energy distribution functions for two OCCN *gauche* conformations, possibly with and without an intramolecular H-bond, are shown in Figure 6. For the HB structure, there is only an inflection point at *E* = −17 kJ/mol, indicating the upper limit of the interactions with the more strongly bound water molecules, which may be act as donors in intermolecular H-bonds to the outer oxygen of the NO_2_ group in structure 9 (Figure 3) The pedf for the NHB structure (**10**) is a well-developed maximum-minimum curve up to *E* = −12 kJ/mol. This shape indicates H-bonds between the freed alcohol OH and the acceptor water molecule(s). The pedfs always show distributions because the thermal disordering does not allow the maintenance of the strongest O (water)…H–O intermolecular bond. The strongest interaction is represented by the onset value of the pedf, *E* = −30 kJ/mol in this case.

2-NC ethanol (2-isocyanoethanol). Only gas-phase studies have been found for the molecule by Møllendal *et al.* [26]. Out of five considered conformers, the OCCN *gauche* structure is the most stable with an (O)H…N distance of 256 pm. The authors characterize this interaction as stabilizing the conformation electrostatically when compared to the two OCCN *trans* forms and two other *gauche* conformations having the intramolecular H-bond disrupted

#### 3.2.2. α-Substituted Carboxylic Acids

α-OH and α-keto acids. The prototype for the hydroxy acids is glycolic acid (α-OH acetic acid). Whereas the O–H…O= type intramolecular H-bond is easily reachable through the formation of the H–O–C–C=O five-member ring in the case of the *syn* conformation for the carboxylic group (see Figure 5), the HO…H–OC=O bond is also conceivable if the carboxylic group adopts the *anti* form. On the basis of the reported OH stretching frequency of 3585 cm^−1^ in comparison with 3682–3684 cm^−1^ in ethanol and methanol, Gu *et al.*, [41] concluded that the red-shift can be attributed to the formation of the structure, where the α-OH is the hydrogen-bond donor to the O= atom of the *syn* –COOH group. No in-solution study has been found for this molecule.

Lactic acid (α-OH propionic acid). Borba *et al.* [170] obtained the FT-IR spectrum for lactic acid in argon and xenon matrices. By performing B3LYP/6-311++G** and MP2/6-31++G** gas-phase calculations, four conformers were identified with observable (>1%) populations. About 92% of the conformers (*T* = 298 K) adopt an eclipsed O=C–C–O moiety, where the alcohol hydrogen is in a weak H-bond with the carbonyl oxygen. Relatively strong intramolecular H-bond is formed between the carboxylic hydrogen and the alcohol oxygen, when the carboxylic group takes the *anti* conformation. It is remarkable that the relative energy including zero point vibrational contribution for the conformer with *anti* –COOH group was calculated at 10–11.4 kJ/mol using the B3LYP/6-311++G(d,p) and MP2/6-31++G(d,p) levels of theory. This value is much smaller than 21.3 kJ/mol calculated by Nagy [79] at the CCSD(T)/CBS level for the *syn-anti* conformational energy difference for acetic acid. The large energy difference, beyond the likely basis set effects, would indicate the considerable stabilization of an *anti* –COOH group in an intramolecular H-bond. The analysis in [170] was limited to the gas phase, no in-solution calculations were performed.

**Figure 7:**
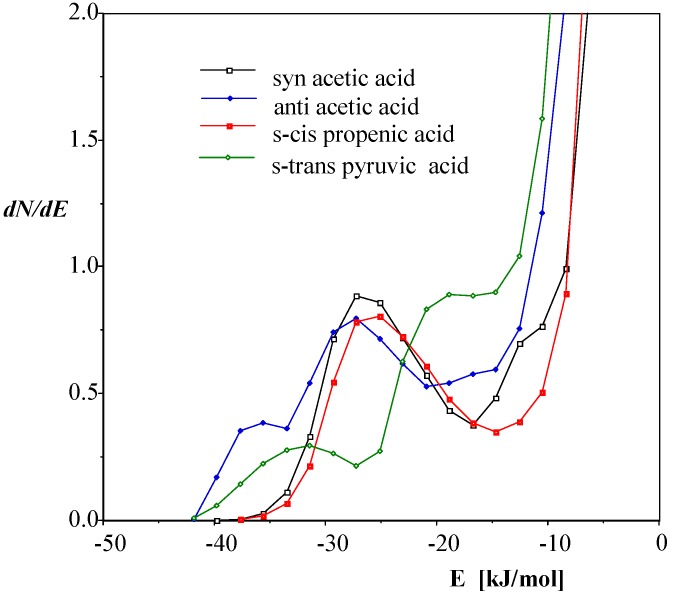
Solute-water pair-energy distribution functions for *syn* acetic acid (**11**); *anti* acetic acid (**12**); *s-cis* propenic acid (**13**); and *s-trans* pyruvic acid (**14**) with structures shown in Figure 5.

Pyruvic acid (α-keto propionic acid) is the simplest α-keto carboxylic acid presenting the *s-cis*/*s-trans* conformational isomerism. This type of structural variation emerges for systems with a double bond-single bond-double bond (DSD) substructure (Figure 7). Dyllick-Brenzinger *et al.* [27] concluded from gas-phase microwave studies that the molecule adopts the O=C–C=O *s-trans* form in its most stable conformation, and there is an intramolecular H-bond between the keto oxygen and the hydrogen of the carboxylic group in its *anti* conformation. Theoretical studies by Yang *et al*., [171] confirmed this conclusion.

In a recent study of DSD molecules by Nagy and Sarver [117], the above, *s-trans*/*anti* –COOH conformation was found as the most stable structure optimized at the B97D/aug-cc-pvtz level in the gas phase. In-solution structural studies were performed by applying the continuum solvent models (IEF-PCM) and by specifying explicit dichloromethane and water solvents in MC/FEP simulations. The solvation favors the *syn* –COOH form both for the *s-cis* and *s-trans* conformers in comparison with the *s-trans*/*anti* –COOH species. The solvent effect is, however, still not enough for stabilizing any *syn* –COOH structure in dichloromethane, but it is enough in water, where the *s-trans/syn* –COOH form is more stable by 2.2–4.6 kJ/mol than the *s-trans/anti* –COOH structure.

Figure 7 below shows some remarkable differences in the pair-energy distribution functions for simple acids. The common conformation is *syn* for a –COOH group without substituents on the aliphatic chain (**11**). The *syn* acetic acid generates a well-resolved maximum-minimum pedf in the −38 to −17 kJ/mol interaction energy range. It must include solute-water interactions with donor waters to the carboxylic oxygens and when the O–H…O (water) bond is formed with the carboxylic hydrogen. This is the most common pattern for –COOH…water intermolecular interactions. It is noteworthy that the three types of interactions overlap in the pedf, creating a single maximum.

The *anti* conformation for the acetic acid carboxylic group (**12**) results in a split maximum. The difference is characteristic, but no study has pointed out yet what interactions belong to the energy range −43 to −33 kJ/mol, and what intermolecular interactions can be characterized by *E* = −33 to −21 kJ/mol. Since the overall pedf is not resolved at *E* = −33 kJ, the “weaker” representative of the stronger interactions still extend beyond −33 kJ, leading to a second elevation of the pedf.

The general shape of the pedf for the *s-cis* propenic acid (**13**) is very similar to that of the *syn* acetic acid. The small differences, less high peak and minimum shifted toward the less negative *E* value, may not be significant under the simulation conditions in [117]. If it is still significant, it may indicate the effect of the one-carbon-longer chain with a double bond *cis* to the carbonyl oxygen.

Pyruvic acid (**14**) again presents a split pedf. The two doubly bonded oxygens mutually affect the charges for each other. No such charge redistribution takes place for the other three structures, thus the α-keto group characteristically modifies the pedf for an aliphatic carboxylic acid. The keto group is a strong H-bond acceptor site, still the first maximum of the pedf is presumably assignable to the C(carboxylic)=O…H (water) intermolecular H-bonds. There are interaction energies for the *anti* acetic acid in the same range, where no competing C=O group exists for this simple acid. An opposite assignment would lead to the conclusion that the hydration of the carboxylic group for pyruvic acid has to be shifted toward an unreasonably low energy range of −27 to −15 kJ/mol.

All these results refer to neutral monomeric pyruvic acid (5%–16% dissociate if forms 0.1–1 molar aqueous solution). IEF-PCM/B97D/aug-cc-pvtz optimizations in dichloromethane and water found that a species composed of two *s-trans/syn* –COOH monomers is the most stable dimeric form with equal geometries for the monomer constituents. Calculations of the potential of mean force curves for the dimerization of pyruvic acid indicate sensitive differences in the pmf whether only monomer charges or solute-solute polarized charges were also used. Charges in the latter case were derived on the basis of the in-solution MEP for the dimer. The dimeric fraction was calculated to be nearly 100% when the solute-solute polarized charges were gradually considered in the C(carboxyl)…C(carboxyl) separation range of 384–484 pm. However, using monomer charges all way down, 51% was calculated for the degree of association in dichloromethane, and only a shallow minimum was obtained in the C…C separation range of 350–650 pm in aqueous solution which corresponds to a low level of solute association [117].

α-Halogen acids. The prototype in this case is α-fluoroacetic acid. Chermahini *et al.*, [172] found four local-energy-minimum structures for this molecule in the gas phase by *ab initio* and DFT calculations. All molecules show slightly distorted C_S_ symmetry, thus the heavy atoms are almost co-planar. In the four molecules, F eclipses the =O or the hydroxyl oxygen, both in the *syn* and *anti* carboxyl conformations. The only arrangement where an intramolecular H-bond can be formed is when the F eclipses the hydroxy oxygen of the *anti* –COOH group. In the most stable conformation, the FCC=O torsion angle is 0° with *syn* carboxylic group. The second most stable form is when the FCC=O torsion angle is 180°. The corresponding MP2/6-311++G** relative energy is 1.7 kJ/mol, and the transition state energy is about 10 kJ/mol. The internally H-bonded conformer (FCCO(H) = 0°) with an *anti* carboxylic group is higher in energy than the most stable structure by 5.4 kJ/mol at the MP2 level. The F…H distance was not reported.

Fluoroacetic acid readily assumes a dimeric form in the gas phase having a *syn* carboxylic conformation. The basis set superposition error corrected dimerization energies were calculated at 47–59 kJ/mol. The dimer with FCC=O about 0° is more stable than the conformer with FCCO(H) of about 0°. The relative energies are 1.8 kJ/mol at the MP2 level. No in-solution studies have been found.

α-Amino acids. Since this family includes the natural α-amino acids, there is an extremely large number of publications available in the literature. This review will survey only the simplest representatives of the family and investigate whether another α-substituent, such as a hydoxymethyl group in serine or a α-hydroxyethyl group in threonine, will remarkably modify the intramolecular hydrogen-bond pattern. This problem emerges only in the gas phase, because natural α-amino acids take a zwitterionic form in aqueous solution.

The simplest molecule of this family is glycine, α-amino acetic acid. In a high-level *ab initio* study, Kasalová [173] calculated the geometry of the two lowest energy conformers of free glycine. The geometries were in good agreement with conformations obtained experimentally and also listed in the paper. The lowest energy structure has C_S_ symmetry (Gly I), and two, presumably weak intramolecular N–H…O= bonds should exist in this conformation. No H…O distances were provided in the paper. The H-bond is supposed to be weak, because the NH_2_ group is a weak H-bond donor. The group is, however, a strong hydrogen-bond acceptor. In the second most stable conformation, Gly II, there is an O–H…N hydrogen bond, which is formed feasibly with the *anti* carboxylic group. Considerably smaller relative energies were calculated for the second stable glycin conformer in former calculations (see a compilation in [173]) because the formation of the hydrogen bond must reduce the internal energy increase due to the *syn* to *anti* transformation of the –COOH group.

The structure is zwitterionic in aqueous solution, which can be derived from both gas-phase conformations. The mechanism of the formation could be, however, interesting. Nagaoka *et al.*, [174] studied the intramolecular proton transfer from the neutral form with *anti* –COOH group into the zwitterion in aqueous solution through molecular dynamics simulations. The authors applied a reactive potential energy function developed on the basis of the empirical valence bond method. The free energy change through the proton transfer was calculated by means of the FEP method. The zwitterion was found to be more stable than the neutral form by 35.4 ± 6.1 kJ/mol at *T* = 300 K. The activation free energy in the process zwitterion to neutral form is 70.4 ± 5.7 kJ/mol. Both values are in good agreement with available experimental values. Tuñón *et al.*, [175] performed a QM/MM molecular dynamics simulation for the intramolecular proton transfer in aqueous solution. The QM part was considered by an (unspecified) DFT functional and the basis set was of double-ξ quality + polarization functions. The overall 2000 fs long simulation protocol indicated the proton transfer in the 250–300 fs time range. The authors predicted that the activation energy may not be too large. In fact, using the computational results of Nagaoka, the activation free energy, starting from the neutral form should be about 35 kJ/mol, which is really not a too large value for a tautomeric reorganization. For example, Lunazzi at al. [176] found a tautomeric proton relocation feasible for triazoles with experimentally measured activation free energies in the range 40–60 kJ/mol.

However, Tortonda *et al.*, [177] considered the “good” estimate for the barrier obtained by Nagaoka to be a consequence of the HF parameterization of the reaction potential. It was stated that such parameterization severely overestimates the activation barrier for this process. Furthermore, the experimental barrier height, in the opinion of Tortonda *et al.*, refers to the interconverison of the in-solution Gly I to Gly II conformations rather than for the Gly II to zwitterions process. The Gly II conformer optimized at the MP2/6-31+G** level in continuum dielectric water solvent model is more stable than the Gly I species by 11.2 kJ/mol [178]. Since the lifetime of Gly II is very small and the Gly I to Gly II conformational change requires a non-negligible barrier of about 46 kJ/mol [175], Tortonda *et al.*, [177] attributed the experimental barrier to the zwitterion formation from Gly I rather than from Gly II. If a water molecule forms a doubly H-bonded bridge between the H_2_N and HOC=O sites for Gly II, the calculated barrier is 65.1 kJ/mol [178]. Then these authors predicted that an intermolecular proton transfer in the indicated arrangement would be unlikely. Nevertheless, the formation of the zwitterion through intermolecular protonation of the neutral amine in Gly I, where the nitrogen lone-pair could instead accept a proton from a neighboring water molecule, remained as a possibility. This would correspond to the Tsuchida-Yamabe mechanism, discussed in Section 3.1.

Correct modeling of the dissolution of serine (α-amino, β-hydroxy propionic acid) in water is a delicate theoretical problem. Gronert and O’Hair [179] theoretically derived 51 serine conformers, and concluded that only a few of them are of small-relative-energy species. In a gas-phase study using the LA-MB-FTMW technique, which combines laser ablation (LA) with molecular beam Fourier transform microwave spectroscopy (MB-FTMW), Blanco *et al.*, [180] identified seven conformers with observable populations. The lowest-energy conformer was similar to Gly I for glycine, with an additional O–H…NH_2_ H-bond by the alcohol hydroxy. In the second lowest energy structure, the *anti* carboxylic group forms a H-bond to the amine, and the alcohol OH acts as a proton donor to the carbonyl oxygen and behaves as a proton acceptor in a N–H…O bond. The substructure resembles Gly II of glycine. Two low-energy structures of Gronert and O’Hair were selected by Tortonda *et al.*, [177] for in-solution studies. For the easier comparison, the H-bond pattern will be characterized as that for the gas-phase glycine and coded as ser (Gly I) and ser (Gly II). The most stable gas-phase serin conformer at the DFT/B3PW91/6-31+G** level is ser (Gly II). This conformer is more stable in enthalpy by 0.9 kJ/mol than ser (Gly I), where the β-OH is only H-bond donor to the nitrogen atom. In solution, the β-OH is only a H-bond acceptor from H–N in ser (Gly II) and is not involved in any intramolecular H-bond in ser (Gly I). Accordingly, the enthalpy of ser (Gly I) relative to ser (Gly II) increases to 15.6 kcal/mol. The zwitterion is more stable in enthalpy than the neutral ser (Gly II) by 8.4 kJ/mol in aqueous solution. The OH group in the zwitterion is free to form intermolecular H-bonds with the solvent. Taking the computational results together, the conclusion was that an intramolecular proton transfer from the ser (Gly II) conformer into the zwitterionic serine species is preferred.

#### 3.2.3. *Ortho* Phenols and Naphthols

In this subsection, both 5- and 6-member rings for forming intramolecular H-bonds will be considered in connection to the phenolic (naphtholic) OH. This author considers comparison of *ortho* phenols more important than strictly maintaining the categorization by the number of ring members. The only noteworthy difference being that the O–H…*X* bond angles could deviate.

Compilations for experimental O–H vibrational frequencies of *ortho* phenols measured in the gas phase or in dilute solutions of low-dielectric-constant solvents [181,182,183] suggest that the O–H…*X* intramolecular H-bond exists in these phases. Appearance of this bond in aqueous solution is a more complicated question.

2-OH phenol (catechol). By interpreting the gas-phase microwave spectrum, Caminati *et al.*, [184] concluded that the structure forms an intramolecular H-bond. However, Mandado *et al.*, [7] did not find a (3, –1) BCP and a related H-bond in their AIM analysis using the B3LYP/6-311++G** electron charge density. Reynolds [185] calculated the relative free energy of the catechol conformers and found that the O–H…O structure with a H-bond (HB) is preferred in comparison to the H–O…O–H disrupted H-bond (DHB) form in aqueous solution.

2-OH benzylalcohol. Kumar *et al.*, [186] recorded the UV, IR and microwave absorption spectra in a supersonic jet. A single conformation was identified, where the phenolic OH is a donor in the intramolecular H-bond to the alcohol oxygen. The authors also assume the existence of a weak O–H…π interaction between the alcohol OH and the aromatic ring on the basis of a second minimum in the spectrum. The two types of intramolecular hydrogen bonds were assigned to absorption in the RIDIR spectrum at 3494 cm^−1^ (O–H…O) and at 3636 cm^−1^ (O–H…π). The minima were reproduced at the M05/cc-pvtz level. A quite different theoretical spectrum was predicted for the conformer where the alcohol OH is the proton donor to the phenolic O. This local-minimum-energy structure is higher in energy than the global minimum by 10.5 kJ/mol at the M05/aug-cc-pvtz level after zero-point energy correction. No solvent effect study was provided.

Simplerer *et al.*, [181] compared the phenolic OH IR-frequencies for *ortho* substituted phenols in dilute CCl_4_ solutions. The experimentally observed red-shift of 202 cm^−1^ for the OH frequency in the 2-OH benzylalcohol methyl ether compared with the pure phenol supports the model that there must be a strong O–H…O intramolecular H-bond in 2-OH benzylalcohol and in their derivatives.

2-Halogen phenol. The gas-phase electron diffraction experiment predicts a mixture of HB and DHB structures for 2-F phenol [187], with preference for the HB structure. 2-Cl phenol forms an intramolecular H-bond in the gas phase and both *ortho*-halogen phenols maintain a HB structure in dilute solutions of low-dielectric-constant solvents [181,182,183]. Recent theoretical studies by Nagy [15,64] confirm this finding: the HB structure is almost exclusive either with a 2-F or a 2-Cl substituent in CCl_4_, and the DHB 2-F phenol fraction was estimated at less than 10% in chloroform. In aqueous solution, the hydration itself favors the H–O…*X* (*X* = F, Cl) DHB structure, but the total relative free energy is still favorable for the HB conformers by about 3–5 kJ/mol, corresponding to at least of 80% HB structure in the equilibrium composition [15].

2-NH_2_ phenol. The term “aminophenol” may be used in a more general sense as regarding the species when the –NH_2_ group is a substituent on the benzene ring for a phenol, or resides on an alkyl substituent connecting to a benzene ring bearing one, two, *etc.*, OH substituent(s). This latter group will be discussed as β-substituted ethylamines in the next section.

Probably due to the high melting point of 174 °C for 2NH_2_-phenol, no experimental gas-phase study has been found in the literature. The neutral (non-zwitterionic) form of 2NH_2_-phenol was studied by Nagy [64] in the gas phase, chloroform and water solvents. In principle, there can exist two intramolecular H-bonds for this molecule, namely O–H…N and N–H…O. The so-called aniline-type-NH_2_ group, as a benzene-ring substituent, is much less basic than an amino group on a saturated chain. The calculated free energy difference is almost zero in the gas phase for the two types of the intramolecular H-bonds. In both solvents the N–H…O bond was found to prevail, although the calculated relative free energies strongly depend on the applied level of theory and the manner of calculating the solvent effects.

2-NO_2_ phenol. Despite the weak hydrogen-bond acceptor character of the –NO_2_ group, the authors of the gas-phase electron diffraction study [188] convincingly argue in favor of the O–H…O(NO) intramolecular H-bond for the isolated molecule. The six-member ring can be conveniently formed. The optimized H…O and O–H…O H-bond parameters calculated at the B97D/aug-cc-pvtz level [64] agree with the experimental values within their respective certainties.

Both in chloroform and water, the theoretical calculations predict a negligible fraction for the DHB conformation with a disrupted intermolecular H-bond. The calculated O–H stretching frequency for the H-bond donor group deviates only by 2 cm^−1^ from the experimental value. The good agreement was considered as an indication of the need for high-level, IEF-PCM/B97D/aug-cc-pvtz geometry optimizations for exploring the relative free energies between HB and DHB conformers in solutions.

2-COOH phenol. The intramolecular H-bond is formed within a six-member ring including the phenolic OH. The molecule may be considered as a β-hydroxy carboxylic acid, as well. Accordingly, it will be compared with the saturated β-hydroxy carboxylic acids in the next section.

The molecule can adopt several conformations, although only one of them is highly populated and was assigned in IR experiments. The spectrum was recorded by Fiedler *et al.*, [189] in tetrachloride solution and indicated a strong intramolecular H-bond. The deviation of the OH stretching frequency from that in phenol was 395 cm^−1^. The theoretically calculated deviation is 359 cm^−1^ at the B3LYP/6-311+G(d,p) level. The lowest-energy conformer is planar, the phenolic OH is a H-bond donor to the carbonyl oxygen of the *syn* carboxylic group. The =O…H distance and the =O…H–O bond angle were calculated at 176 pm and 145°, respectively. The second-most-stable conformer is higher in energy by 14.3 kJ/mol, where the phenolic OH is the H-bond donor to the *syn* carboxylic OH. Similar conclusions were drawn by Yahagi *et al.*, [190] by interpreting the gas-phase IR frequencies of the phenolic OH.

In a former calculation by Nagy *et al.* [45], the two conformers above were found also to be the most stable with MP2/6-31G*//HF/6-31G* energy separation of 13.7 kJ/mol and free energy difference of 12.1 kJ/mol at *T* = 298 K. All other conformers are much higher in free energy, supporting the estimate of Fiedler that the population of the lowest-energy form is 99.7%.

Nagy *et al.*, also investigated if the intramolecular H-bond would be maintained in aqueous solution by performing NpT MC/FEP simulations using the OPLS pair-potential. Comparing the two stable conformers, the solvation itself would favor the conformation with an H–O(carboxyl)…H–O(phenol) bond by 6.1 kJ/mol, but the total relative free energy is still 6.0 kJ/mol in favor of the =O…H–O(phenol) form. Even much larger solvent effect, 30.0 kJ/mol, was calculated in favor of the conformer when the phenolic OH rotates by 180°, thus when the phenolic group is free for hydration. However, the total relative free energy still remains too high by 17.4 kJ/mol at this new geometry when is compared with the most stable one. In conclusion, the conformer most stable in the gas phase with =O…H–O intramolecular H-bond remains as the predominant species in solution, although 8% second-stable form is also expectable in comparison with its gas-phase population calculated at about 1%.

1-NO and 2-NO naphthols. Ivanova and Enchev [191] performed in-solution experimental and theoretical studies for these molecules. Using NMR spectroscopy in CHCl_3_ and DMSO solvents, they found that both structures exist only in the tautomeric =N–O–H oxime form at an observable fraction. This suggests that the relevant structures correspond to 1,2-naphthoquinone monooximes. The theoretical studies at the MP4(SDTQ)/6-31G*//6-31G* level augmented with PCM solvent calculations found an equilibrium between the *syn* and *anti* oximes, although the preferences are different by the two solvents. H-bonds are only possible in the *syn* oxime conformation with the neighboring quinone oxygen. This conformer is favored for the 1-*syn*-oxime-2-naphthoquinone (1-NO-2-naphthol) in both solvents, although the *anti* oxime was also found experimentally and predicted theoretically. For the 2-oxime-1-naphthoquinone (2-NO-1-naphthol), the authors found experimentally that only the *anti* oxime form exists in solution, in contrast to prior experimental results. The calculated barrier for the oxim to nitroso form tautomerization is too high along an intramolecuar proton transfer path, explaining the absence of the 1-NO form.

### 3.3. 6-Member Rings

#### 3.3.1. β-Substituted Ethylamines

**Figure 8:**
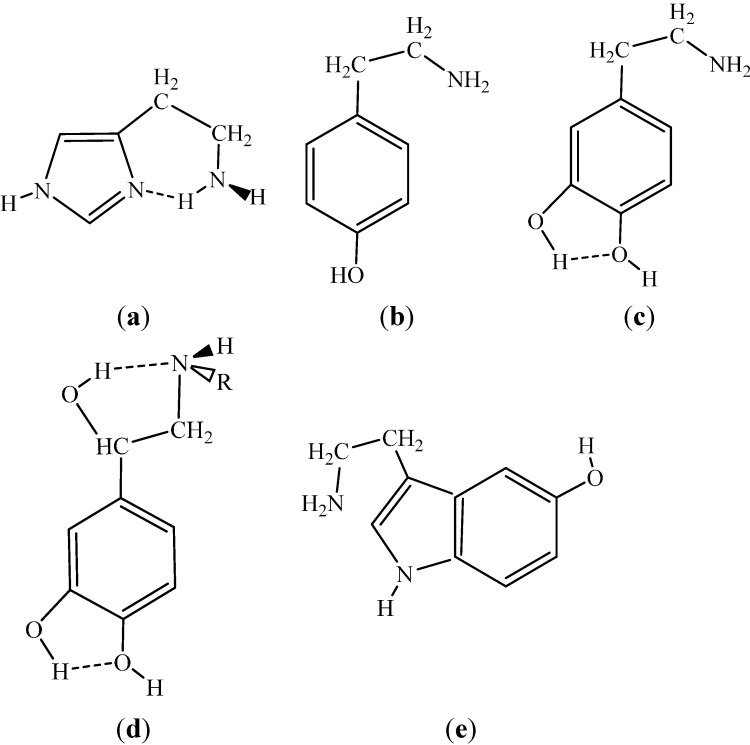
Neurotransmitters portrayed in the neutral form: (**a**) Histamine; (**b**) Tyramine; (**c**) Dopamine; (**d**) Norepinephrine (R=H), Epinephrine (R=CH_3_); and (**e**) Serotonin.

Two members of this family, 2-aminoethanol and ethylenediamine, capable of forming five-member rings for developing an intramolecular H-bond were already investigated in Subsection 3.2.1. Further members of the family with some having aromatic rings in the β-position which allow for H^+^…π interactions with protonated species will be surveyed here (Figure 8). Indeed, these ethylamines belong to the group of the extremely important neurotransmitters, which overwhelmingly adopt the amino N-protonated form at pH = 7.4, where they are involved in biological signal-transduction processes. Nonetheless, neurotransmitters maintain some small percent of the neutral form even at this pH, and create another zero-net-charge species, the zwitterionic structure up to 7.2% in total for the two forms for molecules b-e in Figure 8 [123,192]. These protonation states [193], more abundant at higher pH, will also be discussed below.

For histamine, formation of an intramolecular H-bond between the ethylamine side chain and the N1 nitrogen of the imidazole ring is feasible both in the neutral and the protonated forms. In cases of norepinephrine and epinephrine, an OH group, as another β-substituent is also found in the molecule. For these two latter, O–H…N and N–H^+^…O bridges can be formed in five-member rings for the neutral and protonated species, respectively. Furthermore, an N(amine)–H… π or an N(amine)–H^+^…π intramolecular H-bond is always possible for each molecule in Figure 8. The methods applied to the gas-phase and in-solution structure analyses are summarized in Table 2.

Histamine (2-(1*H*-imidazol-4-yl)ethanamine). Details regarding the challenges posed by the tautomerization of this molecule were provided recently [22]. This review remains focused on aspects associated with the intramolecular H-bond formation. The rotational spectrum of gas-phase histamine was recorded by Vogelsanger *et al.* [194]. Four major conformations were identified, all of which are stabilized by intramolecular H-bonds involving a *gauche* ethylamine side-chain. These conformers encompass the N_1_H–N_3_H tautomerization for the imidazole ring and both H-bond donor and acceptor properties of the imidazole as well as of the amino group. For protonated histamine, two major gas-phase isomers were detected by Lagutschenkov *et al.*, in the IR spectrum [195]. The more stable one is protonated on the ring and a N_1_(ring)-H^+^…N(amine) H-bond is formed. This structure is more stable by a few kJ/mol than the N_1_…^+^HN(amine) state formed by proton jump from the ring to the amino group. Both protonation forms can preferably create H-bonding within a *gauche* ethylamine side-chain conformation.

In this paper, Lagutschenkov *et al.*, provided an excellent overview of the present status of histamine-structure research. The histamine problem is threefold: Protonation state, prevailing tautomer and conformations. If the neutral and the monoprotonated structures are separately investigated, the problem becomes twofold. The amine p*K*_a_ is 9.75–9.80, so neutral histamine must be the major species in aqueous solution when it is dissolved in pure water. The solute becomes protonated only to a very small degree under such conditions, but then becomes the prevalent species at pH = 7.4.

**Table 2:** Comprehensive summary of the applied experimental and theoretical methods for the conformational/tautomeric equilibria of neurotransmitters in aqueous solution ^a^.

Structures	Theor. Calc.	Gas-Phase	Aqueous Solution					
	Theor. Ref.	Exp. Ref.	*E*_int_	ZPE/G_th_	Cont. Solv.	MC/FEP	MD/FEP	Exp. Ref.
Histamine neutral		[194]						
	[196]		MP2/6-31G		SCRF			[197]
	[198]		MP2/6-311++G**	+		OPLS		
	[199]		B3LYP/6-311G**		SCRF			
	[200]		HF/6-31G*		PCM			
	[201]		MP2/augccpvtz	+	MST ^b^			
Protonated		[195]						
	[196]		MP2/6-31G		SCRF			[197]
	[198]		MP2/6-311++G**	+		OPLS		
	[200]		HF/6-31G*		PCM			
	[202]		HF/6-31G*				Amber	
Tyramine neutral		[203]						
	[123,204]		MP2/6-31G*		PCM	OPLS		
Zwitterion	[123,204]		B3LYP/6-311++G**		PCM	OPLS		[204]
Dopamine neutral		[205]						
	[206]		AM1		SM1 ^c^			[204,207]
	[123]		B3LYP/6-311++G**		PCM	OPLS		
Dopamine zwitterion								
	[123,204]		B3LYP/6-311++G**		PCM	OPLS		[204]
Protonated		[208]						
	[208]		AM1		SM1			[204,207]
	[209]		HF/6-31G*		PCM	OPLS		
Anionic	[206]		AM1		SM1			[207]
Norepinephrine neutral		[210]						
	[211]		MP2/6-31G*		PCM			[207]
Protonated	[192]		MP2/6-31G*	+		OPLS		[192,207]
	[211]		MP2/6-31G*		PCM			
Epinephrine neutral		[212]						
Protonated	[213]		B3LYP/6-311++G**	+	IEFPCM ^d^			[204,207]
Serotonin neutral		[214]						[204]
Protonated		[215]						
	[216]		MP2/6-31G*	+	IEFPCM	OPLS		[192,204]
	[217]		MP2/6-31G*	+	IEFPCM			

^a^ In cases, when more than one quantum mechanical methods were used, the level producing the best result is indicated. The “+” sign indicates that ZPE/G_th_ was calculated; ^b^ Reference [57]; ^c^ Reference [59]; ^d^ Reference [52].

Calculations to date show no consensus regarding the structure of the neutral histamine in aqueous solution. Karpińska *et al.*, [196] using the continuum solvent SCRF method at the MP2/6-31G//MP2/6-31G level found the N_3_H tautomer as the most stable without an intramolecular H-bond. The next stable structure is higher in energy by only 0.8 kJ/mol, where the ring proton of the N_1_H tautomer is the H-bond donor to the amino group of the *gauche* ethylamine side chain. Nagy *et al.*, [198] found the N_3_H tautomer with predominantly (83%) *trans* ethylamine side-chain conformation as the most stable structure on the basis of MP2/6-311++G**//HF/6-31G* + MC/FEP calculations. This structure prevents the formation of an intramolecular H-bond in an aqueous solution. The second most stable form, N_3_H/*gauche* ethylamine chain, is present at 12%.

In contrast to the above results, Ramirez *et al.*, [199] predicted the N_3_H/*gauche* side-chain conformation to be predominant at the PCM/B3LYP/6-311G** level. Raczyńska *et al.*, [200] performed theoretical calculations utilizing the PCM approach at the HF, MP2, and B3LYP levels with basis sets up to 6-311++G**. The prediction is a *gauche-trans* equilibrium for the neutral N_1_H tautomer in aqueous solution. Forti *et al.*, [201] published recently a paper describing a multilevel strategy for the exploration of the conformational flexibility of small molecules. The predicted *trans/gauche* ratio strongly depends on the applied basis set, changing from 64/36 to 48/52 when the B3LYP/6-31G*, MP2/aug-cc-pvdz and MP2/aug-cc-pvtz theoretical levels are considered. On the other hand, the N_1_/N_3_ ratio was predicted consistently as 48:52. In summary, structure predictions for the neutral histamine in aqueous solution have not been able to form a consensus.

The agreement is much better regarding the monoprotonated species [196,198,200,202]. All these calculations favor the N_3_H tautomer in combination with a protonated ethylamine side-chain. When the side-chain conformation was also investigated, the predominant *gauche* structure forms an intramolecular N_1_…^+^HN(amine) H-bond. Thus the stability order differs from that in the gas phase where this species is only the second most stable structure.

An experimental conformational analysis in aqueous solution was performed by Kraszni *et al.* [197]. These authors determined the position-specific standard ^1^H NMR coupling constants for the *gauche* and *trans* conformers. This study helped predict the population of the *trans* conformer to be 41%, 38%, and 50% in neutral, monocationic and dicationic forms, respectively.

Tyramine (4-(2-aminoethyl)phenol). A good summary of recent gas-phase experiments for studying the structure of ethylamine derivatives with β-phenol and catechol substituents was provided by Ishiuchi *et al.* [218]. For tyramine, the gas-phase conformations were determined by Melandri and Maris [203] using a free-jet microwave study. The authors found four structures where the side-chain adopts the C(ring)–C–C–N *gauche* conformation and where an N–H…π H-bond may stabilize the structure. The four structures differ in the relative rotational positions of the –NH_2_ and the OH groups. MP2/6-31G* calculations predicted an energy range of 1 kJ/mol for these conformers. With a *trans* side-chain, the lowest relative energy is 5 kJ/mol.

Nagy *et al.*, [123,204] studied tyramine theoretically in the gas-phase and in aqueous solution. Although the conformer energies of the neutral form with *gauche* and *trans* chains hardly differed as calculated at the B3LYP/6-31G* level, the free energy at *T* = 298 K was lower by 1.8 kJ/mol for the *trans* structure, in contrast to experimental data. Tyramine may form an N–H…π intramolecular H-bond in the case of the *gauche* side-chain conformation. The B3LYP method is known to fail accounting for dispersion interactions. On this basis, one may think that the lack of considering dispersion interactions in [123] led to the prediction of the absence of an intramolecular H-bond, which would have been stabilized by favorable and remarkable dispersion interactions otherwise. Not accounting for dispersion interactions may be the major the reasons for the *trans* preference, because these kinds of interactions are more important between the NH_2_ group and the ring than their role in stabilizing an intramolecular H-bond. The distance between the amino group and the ring is smaller in the *gauche* than in the *trans* conformation; consequently a missed account of dispersion contributions to the conformer stabilizing energy terms would more sensitively affect the *gauche* than the *trans* form. Indeed, MP2/6-31G* calculations by Melandri and Maris above, where the dispersion interactions are considered, clearly indicate the *gauche* preference.

In aqueous solution at physiological pH 7.4, the protonated form is present at about 99% [204]. For this structure, the only stabilization possible is when the –CH_2_–CH_2_–NH_3_^+^ chain bends above the aromatic ring, forming an N–H^+^…π intramolecular H-bond. Also in the solution are two zero-net charge forms, zwitterionic (zw) and neutral (neu), in a total population slightly more than 1%. The zw: neu ratio rapidly decreases from 10.72 to 2.45 in aqueous solution when the temperature is raised from 14 to 37 °C [123]. Nonetheless, the zw form must be the prevalent zero-net-charge structure when tyramine dissolves in pure water at room temperature. Theoretical calculations at the PCM/MP2/6-31G*//B3LYP/6-31G* level found the neu form prevalent. In contrast, using the MC/FEP method, the zw form is the stable overall neutral tautomer, although the relative free energy is much exaggerated in comparison with the derivable experimental value [123].

Dopamine (4-(2-Aminoethyl)benzene-1,2-diol). Cabezas *et al.*, [205] found experimentally seven conformers for the gas-phase (neutral) dopamine. All structures maintain an O–H…O H-bond on the benzene ring. The ethylamine side-chain has a *gauche* C(ring)–C–C–N conformation. The seven structures come into existence with N–H…π interactions in different rotational positions of the –NH_2_ group relative to the O–H…O bond. Lagutschenkov *et al.* [208] recorded the gas-phase IR spectrum for protonated dopamine. Not surprisingly, the protonated side-chain in the most stable conformers adopts the *gauche* arrangement as defined above for the neutral form and bends above the aromatic ring. This interaction corresponds to a N–H^+^…π intramolecular H-bond. The authors performed B3LYP and MP2 calculations using the cc-pvdz basis set and concluded that the H-bond on the ring is of O(3)–H…O(4) type in the two lowest energy structures, separated only by 0.1 kJ/mol in free energy.

Other theoretical studies in the literature also found a H-bonded 3-OH/4-OH substructure, nearly coplanar with the ring both in the gas phase and aqueous solution, see, e.g., [123,206,209]. Šolmajer *et al.*, [207] studied the protonation process experimentally for several neurotransmitters, including dopamine. The protonated form stably exists up to about pH = 8 and then the proton is gradually lost in the pH range of 8–10. Less than 5% of the amines remain protonated above pH = 11.5. The ethylamine side-chain may adopt three main conformations along the C(ring)–C–C–N path. Two, nearly equal-energy *gauche* and one *trans* conformations are stable, with different rotational positions for the neutral –NH_2_. An intramolecular H-bond in the form of N–H…π or N–H^+^…π is possible only in the *gauche* conformation of the side-chain.

Both the neutral and a zwitterionic zero-net-charge structures are present in aqueous solution, where one of the hydroxy protons jumps to the –NH_2_ group or (more likely) the zwitterion gets formed by water catalysis. Although the population of the zero-net-charge form is pH dependent and is present in a total of only about 3% at pH = 7.4, the neu:zw ratio of about 10 must be constant in any aqueous solution [204]. By performing MC/FEP solvent-effect calculations, Nagy *et al.*, [123] predicted that the proton comes from the 4-OH group in the zwitterion, still maintaining the O(3)–H…O(4) intramolacular bond on the benzene ring. The related conformation of the ethylamine side-chain is preferably *trans*. For the protonated dopamine, the ratio of the *gauche* (G) and *trans* (T) side-chains in aqueous solution was calculated by Nagy *et al.*, [209] theoretically, using HF/6-31G* relative internal energies and MC/FEP simulations for estimating the solvent effect. The predicted G:T ratio of at least 75:25 is somewhat comparable with the experimentally found value of 58:42 at pH = 7 [207].

Norepinephrine (4-[(1*R*)-2-amino-1-hydroxyethyl]benzene-1,2-diol), is a derivative of the 2NH_2_-ethanol with a 3,4-dihydroxyphenyl substituent at C_1_ of the ethane chain. The gas-phase structure of the neutral norepinephrine (with older name: Noradrenaline) was studied by Snoek *et al.* [210]. The authors found that almost the entire population of jet-cooled noradrenaline adopts a conformation with extended ethanolamine side-chain allowing for a H-bond between the side-chain OH and the amino group, as well as between the phenolic hydroxyls.

The prevailing structure is the protonated form in physiological systems, 92.8% at pH = 7.4 [192]. An N–H^+^…O intramolecular H-bond is favorable, which can exist in one of the C(ring)–C–C–N *gauche* conformations and in the *trans* form. In the other *gauche* conformation, where the formation of the N–H^+^…O bond is prevented because of the local O–C–C–N *trans* arrangement, the possible conformer-stabilizing effect through the N-H^+^… π interaction should be emphasized. The two phenolic OH groups form a hydrogen bond like in the gas phase, but probably only on the basis of the distance criterion, since Mandado *et al.*, [7] did not find a (3, −1) BCP for 1,2-dihydroxybenzene.

The equilibrium conformer fractions were calculated at the *ab initio* and DFT levels using the PCM continuum solvent approach and the FEP method in MC simulations [192]. The method applied for calculating the relative internal free energies affect the final conclusions. Overall, the internally bound OCCN conformers were found to dominate the composition in fair agreement with experimental findings at pH = 7 [207].

Using the PCM method, Alagona and Ghio [211] studied the conformer population for neutral and protonated norepinephrine in aqueous solution at the HF/6-31G* and MP2/6-31G*//HF/6-31G* levels. The calculated T fraction regarding the C(ring)–C–C–N torsion of the neutral form is 61%–72%, whereas the *trans* form was populated experimentally by about 59% (pH = 11.5). The T:G ratio for the protonated species was calculated as 89:11 and 44:56 at the HF and MP2 levels, respectively, in comparison with the experimental composition of 65:35 at pH = 7.0 [207].

Epinephrine ((*R*)-4-(1-Hydroxy-2-(methylamino)ethyl)benzene-1,2-diol). Epinephrine (with its older name, adrenaline) is the N-methyl derivative of norepinephrine. Its gas-phase structure was studied by a combination of mass-selected ultraviolet and infrared holeburn spectroscopy [212]. The identified conformation has an extended side-chain structure with an intramolecular O–H…N H-bond. The authors also identified experimentally the H-bonded substructure for the two phenolic OH groups.

Epinephrine is only slightly soluble in water and alcohol, but is readily soluble in aqueous solution of mineral acids. At pH = 7.4 only the protonated form, 94.8% [204], should be the subject of theoretical calculations. Alagona and Ghio [213] studied the conformational equilibrium for protonated adrenaline in aqueous solution at the DFT and *ab initio* MP2 levels and using the IEF-PCM solvation approximation. The C(ring)–C–C–N *trans* arrangement (corresponding to a *gauche* OCCN arrangement) was found as the most stable conformation allowing for the O…^+^H–N H-bond but preventing the H^+^…π interaction with the catechol ring. The two OH groups on the benzene ring maintain the O–H…O H-bond in aqueous solution.

The OCCN *trans* conformation was found to be only 10% in the experimental composition at pH = 9 for norepinephrine [207]. The corresponding value for ephedrine is 13%. Ephedrine is structurally related to epinephrine, bearing an *N*-methyl group, as well, but missing phenolic hydroxy groups and with an additional methyl group on the ethyl chain. Overall, the H-bond pattern for epinephrine is expected to be similar to that for norepinephrine.

Serotonin (5-Hydroxytryptamine). LeGreve *et al.*, [214] investigated the gas-phase serotonin conformers using different spectroscopic methods. They identified eight neutral serotonin conformers including the side-chain both in C(ring)–C–C–N *gauche* and *anti* (*trans*) conformations, and two main rotational positions for the OH group. The most populated conformation is Gpy(out)/*anti* OH, where the *gauche* side-chain is on the pyrrole side of indole, one of the NH_2_ hydrogens points toward the pyrrole nitrogen, and the indole (N)H is in *anti* position with respect to the hydroxy hydrogen. Thus the intramolecular H-bond is basically of an N–H…π type.

Lagutschenkov *et al.*, [215] recorded the gas-phase IR spectrum of protonated serotonin. They found a *gauche* conformation for the protonated ethylamine side-chain rotated toward and above the phenolic ring of the indole moiety. In this position, N–H^+^…π, cation…π interaction stabilizes the structure. The preference of this conformation was supported by B3LYP and MP2 calculations.

Nagy *et al.*, [192] calculated the distribution of the zero-net-charge forms, neutral (neu) and zwitterionic (zw), for serotonin at pH = 7.4. In that solution, the protonated form is present at 99.7%, and the zero-net-charge form is present only at about 0.3%. However, since the determined neu:zw ratio of about 1.2 is pH independent, if serotonin is still non-protonated when dissolved in aqueous solution, the above neu:zw ratio should hold for the major zero-net-charge protonation state.

Alagona and coworkers studied the conformational equilibrium for the protonated serotonin in aqueous solution [216,217]. According to the covalent structure of the molecule, the only possible intramolecular H-bond is of N–H^+^… π type, similar to that for tyramine and dopamine (not considering the stably maintained O-H…O bond for the latter.) For such systems, the correct prediction of the *gauche-trans* conformational equilibrium for the side-chain is crucial. For histamine, norepinephrine and epinephrine, the side-chain conformation is probably more effectively dictated by the possible formation of N–H^+^…N and N–H^+^…O H-bonds.

The serotonin study in [216] shows almost all computational difficulties emerging throughout the conformational analysis for solutes. The obtained relative internal energy results depend on the level of theory used during the calculations. Contributions of the relative thermal corrections to the total relative conformational free energies are critical. It has also revealed that the solvation method, thus whether the relative solvation free energy was calculated at the PCM level or in a MC/FEP process, has an effect on the final results. Should the counterion be allowed to freely move in the solution in MC simulations, or a fixed solute-counterion separation is acceptable for expediting the FEP calculations? Are atomic charges more preferable from CHELPG or RESP fit to the in-solution MEP?

Due to the listed problems, the results from the above study were not conclusive. Different combinations of the relative free energy components, calculated on the basis of the IEF-PCM/B3LYP/6-31G* and IEF-PCM/MP2-6-31G*//B3LYP/6-31G* levels for the internal terms and using MC/FEP relative solvation free energies, could lead to the preference for either the *trans* or *gauche* side-chain conformations. In the absence of experimental data, the “best” choice was not clear. Nonetheless, all calculations predicted an observable equilibrium between *gauche* and *trans* side-chain conformations, since the total relative free energies were within a range of about 4 kJ/mol.

By performing an IEF-PCM analysis for the solution phase, Alagona and Ghio [217] studied the protonated serotonine conformations in the gas phase and in water solvent. The potential curve at MP2/6-31G* level in the gas phase for the hydroxy hydrogen rotation shows that the hydrogen atom is *syn* to the indole (N)H. The IEF-PCM/MP2/6-31G*//MP2/6-31G* free energy in solution is more negative by 5.3 kJ/mol for the *gauche* conformer with the –NH_3_^+^ group rather away (G1) than toward (G2) the indole ring. The *trans* form is higher in free energy by 1.5 kJ/mol than G2. These calculations predict the overwhelming presence of the two gauche conformers in aqueous solution in comparison with the *trans* structure.

In conclusion, the intramolecular H-bond in aqueous solution is generally maintained either in the form of NH^+^…*X* (*X* = O, N) or through NH^+^…π interactions for neurotransmitters with a protonated amino group. For the neutral structures in this phase, the theoretical calculations have led to different conclusions. The neutral form is prevalent generally at pH > 9, where all studied neurotransmitters possessing at least one phenolic OH can adopt also the zwitterionic form in aqueous solution. The pH independent neu:zw ratio is largely varying at *T* = 298 K, from about 0.2 for tyramine to 1.2 for serotonine and to about 10 for dopamine.

#### 3.3.2. β-OH Carboxylic Acids

2-COOH phenol. Salicylic acid is the classical β-OH carboxylic acid in the aromatic series. It was already discussed in Section 3.2.3 as a phenol derivative. The primary structural difference between an aromatic and an aliphatic β-OH carboxylic acid is that the heavy atoms are coplanar for the aromatic molecule unless a neighboring substituent forces the –COOH group to rotate out of the benzene plane. In the case of a coplanar skeleton like 2-COOH phenol, both =O…H–O and O–H…O–H intramolecular H-bonds are conceivable. For the latter type, the donor hydrogen can come from either hydroxy group.

Aliphatic acids. In contrast to aromatic systems, the C(carboxylic)CCO moiety would generally adopt a (nearly) *gauche* or *trans* conformation in the aliphatic series. Unfortunately, no calculations analyzing structure were found for the simplest β-hydroxy carboxylic acid, namely β-hydroxy propionic acid. Its α-amino derivative, serine (α-amino, β-hydroxy propionic acid) was studied as an α-amino acid above. Seven low-energy conformers of l-threonine ((2*S*,3*R*)-2-amino-3-hydroxybutyric acid), the β-methyl derivative of serine, were identified in the gas phase by Alonso *et al.* [219]. In the lowest energy conformation, the alcohol OH is a proton donor to the NH_2_ group, as it was found for 2-NH_2_ ethanol. This bond clearly does not exist for a simple β-hydroxy acid. In the second lowest energy structure (34 cm^−1^, 0.4 kJ/mol above the minimum, as calculated at the MP2/6-311++G** level) the alcohol OH forms a H-bond to the carbonyl oxygen of the *anti* carboxylic group. Because the NH_2_ group also serves as a competing H-bond acceptor, it did not reveal whether an O–H…O= bond is also feasible to the *syn* –COOH group. Geometry results indicate, however, that this interaction could easily come into existence.

A combined spectroscopic and in-solution quantum chemical investigation was carried out by Quesada-Moreno [220] at pH = 1.00, 5.70 and 13.00 in aqueous solution, and the protonation states of the molecule were modeled theoretically under the experimental conditions. The conformational search found 9 zwitterions, 27 anions and 52 cations at the B3LYP/6-311++G(d,p) level of theory, whereas the most stable conformers were optimized at the M062X/6-311++G(d,p) and MP2/6-311++G(d,p) levels of theory, as well. The solvent effects were calculated by means of the IEF-PCM method. As the authors write: “With regard to the zwitterion, the importance of the analysis of the low frequency region (700–30 cm^−1^) in the Far-IR spectra should be noted, because it provides relevant information that can be used to determine the presence of the most stable structures.”

Discussion of the large number of conformations is beyond the possibilities of this review. Regarding the possible H-bonds between the carboxylic and OH groups, conformers of the protonated species may be informative. For this species the –COOH group is not ionized. The presented, low energy conformations are dominated, however, by –NH_3_^+^…O hydrogen bonds (sometimes to two oxygens at the same time) and perhaps only higher-relative-energy conformers would show H-bonds between the carboxylic and OH groups. Related geometries are not provided in the paper, and the reader is advised to turn to the authors directly.

#### 3.3.3. β-NH_2_ Carboxylic Acids

Structural results have been found as prototypes for the aliphatic and aromatic β-NH_2_ carboxylic acids. On the basis of the rotational spectrum for β-amino propionic acid (β-alanine), McGlone and Godfrey [221] concluded that there are two conformers in the gas phase which are similar to those assigned for glycine as conformers I and II and correspondingly to α-alanine. This means that the systems are not sensitive to whether an intramolecular H-bond is formed in a five-member ring (glycine, α-alanine) or in a six-member ring as for β-alanine. Sanz *et al.*, [222] found two more conformers, where the symmetrical Gly I-like form is disrupted into two, non-symmetrical structures with different N–H…O= bonds. A fourth conformation was identified as stabilized by an *n*→π * interaction involving the nitrogen lone pair and the π * orbital of the carbonyl group.

The in-solution structure of β-alanine was recently studied by Nagy [89] in water and chloroform solvents. The α-amino acids are known to take predominantly the zwitterionic form in aqueous solution. Nagy investigated whether the NH_3_^+^…^−^OCO form is also stable in aqueous solution, or if the proton jumps over onto the carboxylate group in the *gauche* NCCC conformation. Calculating the relative internal free energy of the conformers/tautomers at the B97D/aug-cc-pvtz level, and determining the relative solvation free energies in a MC/FEP process, the conclusion was that the *gauche* zwitterion is more stable by about 4 kJ/mol in aqueous solution than the *gauche* neutral form with an *anti* –COOH group forming an H_2_N…HOC=O intramolecular H-bond. The *gauche* zwitterion is more stable than the *trans* conformer by 24–33 kJ/mol.

Optimizing the geometry of the *gauche* zwitterion in chloroform, the starting geometry was chosen as the in-water optimal structure. Through the IEF-PCM/B97D/aug-cc-pvtz optimization, the extra proton from the NH_3_^+^ group returned to the carboxylate group in an *anti* conformation, and the corresponding *gauche* neutral structure was formed. This conformer is more stable by about 17 kJ/mol than the extended *trans* conformer. The *trans* zwitterion is also a local energy minimum on the potential surface, but is higher in free energy by about 58 kJ/mol than the corresponding neutral form.

2-NH_2_ benzoic acid (anthranilic acid). Upon the interpretation of the electronic and IR results for anthranilic acid in a supersonic jet [223], the existence of two anthranilic acid conformers was predicted in the gas phase. The amino group serves as a H-bond donor in both. The carbonyl oxygen is the acceptor in the more stable rotamer, whereas the N–H…O-H bond is formed in the less stable structure. Both bonds must be weak due to the weak acidity of an aniline-type –NH_2_ group.

For studying the solvent effect on the molecular structure, Abou-Zied *et al.*, [224] recorded the absorption and fluorescent spectra of the molecule in neat and binary solvents of varying polarities and H-bonding strengths including cyclohexane, dioxane, acetonitrile, methanol, ethanol and DMSO. By titration in aqueous solution in the pH range of 2–12, the authors derived the p*K*_a_ to be 4.50. They concluded that the intramolecular H-bond pattern, as found in the gas phase, is maintained and that the –NH_2_ group still serves as an intramolecular H-bond donor. The carboxylic group can become a member of a cyclic system where a network of three water molecules forms a bridge between the carbonyl oxygen and the acidic hydrogen. The first and third water of the network act as an intermolecular H-bond donor and acceptor to the carbonyl oxygen and the hydroxy hydrogen, respectively. The trihydrate structure was predicted on the basis of B3LYP/6-311++G(2d,p) calculations.

In an unpublished study, Nagy found that the zwitterionic form is not stable for this molecule in neutral water. Starting from a reasonable zwitterionic form for B97D/aug-cc-pvtz geometry optimization using the IEF-PCM continuum dielectric solvent model, the proton of the –NH_3_^+^ group jumped to the carboxylate group resulting in a O–H…NH_2_ intramolecular H-bond. In the light of the above results, such a structure with an *anti* –COOH group for anthranilic acid, even if exists in aqueous solution, must be a higher-energy conformer. Furthermore, Abou-Zied and coworkers raised the possibility of acid dimerization, which in combination with their conclusions regarding the aqueous solution structure, is more likely in non-protic solvents, e.g., in cyclohexane. The dimeric structure can come into existence favorably with the *syn* –COOH group for a participant.

#### 3.3.4. Cyclic Enols

Due to the electron withdrawing effect of the oxygen atom in a C=O double bond, the hydrogen(s) on the neighboring carbon atom become(s) more acidic. As a consequence, an equilibrium emerges between the –CH*_x_*–C(*R*)=O and the –CH_(*x*−1)_=C(*R*)–OH structural forms. The equilibrium is called keto-enol tautomerism. If there are no additional polar groups in the molecule, at least not close to the carbonyl group, the equilibrium is generally shifted toward the –C=O containing structure. A more complicated situation comes into existence if two carbonyl groups are separated by a CH*_x_* (*x* = 1,2) unit. Typical examples are β-diketones and dialdehydes, β-keto carboxylic acids and esters, the dicarboxylic malonic acid and its esters. For such species, the keto-enol tautomerism was demonstrated experimentally by Moriyasu *et al.*, in different polarity solvents [225]. The determined equilibrium compositions indicate that the preference of the enol structure increases in more dilute solutions but decreases with the increasing polarity of the solvent. For ethyl acetoacetate (for the gas-phase molecular structure of the methyl acetoacetate, see [226]), which is favorably used for syntheses of ketones and carboxylic acid esters, the keto form predominates in chloroform and more polar solvents. In contrast, the enol form is much more favored in all studied solvents but water for acetylacetone. With aromatic rings connected to the β-diketo moiety, the enol form is overwhelming in any studied organic solvents.

Grabowski [2] referring to some former papers emphasized the importance of the interrelation between π-electron delocalization and H-bonding. For molecules possessing a β-diketo moiety, a favorable and coplanar six-member intramolecular H-bond can occur. The situation is similar to the case when the phenolic OH forms a H-bond to the carbonyl oxygen in 2-COOH phenol (salicylic acid). Whereas the formation of the intramolecular H-bond within a six-member ring does not need a solvent-affected keto-enol tautomeric shift for salicylic acid, formation of a H-bond donor hydroxy group is solvent dependent for a β-diketo moiety.

Malondialdehyde. This molecule is the simplest 1,3-dicarbonyl species, actually a dialdehyde. The gas-phase microwave spectrum was recorded by Baughcum *et al.*, [227] and the IR spectrum by Seliskar and Hoffmann [228]. The string “malonaldehyde (3-hydroxy-2-propenal)” in the title of the Buaghcum paper is noteworthy. The authors want to emphasize in the title that the system is subject to keto-enol tautomerism.

For β-dicarbonyl systems, a conjugated double-bond moiety comes into existence in the form of HO–CH=CH*_x_*–C=O (*x* = 0, 1) when the enol structure is created. Such molecules are subject to *s-cis*/*s-trans* conformational equilibrium about the formal single CH*_x_*–C bond. This type of conformational equilibrium was recently studied by Nagy and Sarver [117], who also investigated the effect of the non-polar solvents and water on the in-solution conformer composition. Structures **13** and **14** are examples for the *s-cis* and *s-trans* conformations, respectively.

An intramolecular H-bond can be formed only in the *s-cis* conformation of the enolic malondialdehyde. If the heavy atom skeleton is not entirely coplanar, the structure is called *gauche*, as found for the second stable form of 1,3-butadiene. The above experimental studies found fully planar molecular structure for 3-hydroxy-2-propenal, as the enol form was called in [227]. Earlier theoretical studies have been summarized by Grabowki [2] on the keto-enol tautomerism and the geometric consequences of the process.

If the molecule has a C_2*v*_ symmetry for the planar malondialdehyde in the dicarbonyl form or C_2_ for acetylacetone, then two equivalent enol forms can be derived from the structure. Since the molecules are undistinguishable except, e.g., if there are different isotopes for the oxygens, the proton relocation to one or to the other oxygen cannot be noticed macroscopically. A possible reaction route is that the two OH groups are formed via the intermediate formation of the dicarbonyl structure.

The authors of the experimental studies [227,228] argue, however, in favor of a tunneling mechanism. The two, undistinguishable enol forms, which are now unsymmetrical, could transform into each other through a structure of C_2v_ symmentry, where the electrons of the two double bonds are delocalized. The quantum chemical explanation for the intramolecular proton relocation rests on the acceptance of a double-well potential for the process, where the isoenergetic enol forms correspond to local energy minima, and the symmetrical intermediate structure with four delocalized electrons in a six-member ring correspond to a transition state.

In-solution NMR investigations were performed in chlorofom by Bothner-By and Harris [229] and by Bertz and Dabbagh [230]. Bothner-By and Harris compared a number of *s-cis* and *s-trans* conformational/tautomeric isomers, Bertz and Dabbagh listed former publications in different solvents. These studies reveal that the *trans* enol form of malondialdehyde (called simply *trans*) exists in water, protic and polar solvents, whereas the enol adopts the *cis* form in non-polar solvents. Bertz and Dabbagh found, however, that polar impurities like methanol affect the conformer ratio in CHCl_3_. The methoxy oxygen of methanol competes with the carbonyl oxygen in forming an intermolecular *vs.* intramolecular H-bond with the OH group. This is exactly the problem addressed in the title of this review. Bertz and Dabbagh did not consider an O–H…O intramolcular H-bond as a decisive factor in stabilizing the *cis* conformation.

George *et al.*, [231] optimized six planar conformers for β-hydroxyacrolein (malondialdehyde enol) and compared the energies of the most stable *cis* and *trans* forms at the 4–31G level. They concluded that the most stable *cCc* conformation can create an intramolecular H-bond. In the opinion of Bertz and Dabbagh, the result in favor of the intramolecularly bound structure is not convincing enough, because possible intermolecular H-bonds with proton acceptor molecules, like methanol, have not been considered. Indeed, pointing out the disruption of the intramolecular bond in solution, consideration of gas phase hydrates/solvates would still not be enough; explicit solvent or at least supermolecule studies in continuum solvents should be performed.

The structures presented by Bothner-By and Harris show a number of various conformers/tautomers, which could be in equilibrium in chloroform. By considering a series of solvents listed by Bertz and Dabbagh, chloroform represents a borderline solvent between very low dielectric constant solvents like hexane and carbon tetrachloride *vs.* protic, highly polar solvents like methanol and water. Although Bothner-By and Harris predicted a prevalent *s-trans* conformation, other researcher argue in favor of the *s-cis* form (see for references in [230]). The conformational problem becomes even more complicated if considering that not only *s-cis*/*s-trans* conformers have to be compared, but there are two different arrangements of the OH group relative to the –CH=CH–CH=O moiety.

In summary, prediction of the enolic malondialdehyde conformational/tautomeric equilibrium presents a very complicated structural problem, as revealed from experiments for solutions in moderately polar solvents. Satisfactory high-level theoretical calculations for any in-solution equilibrium, which could make at least initial suggestions about the structural preference have not been found through the literature search.

Acetylacetone. This molecule is the classical target for theoretical considerations of the keto-enol tautomerism. Belova *et al.*, [31] found from gas-phase electron diffraction investigation 100% of the enol form at 300 K and 64% at 671 K. The enol form with C*_S_* symmetry possesses a strongly asymmetric intramolecular H-bond in the gas-phase. For aqueous solution, the C*_S_* symmetry, involving coplanar heavy atoms, has been called in question by Bothner-By and Harris [229], who mentioned that the NMR spectrum reflects the average of two, isoenergtic, non-planar molecular structures in water. The keto form is of C_2_ symmetry. Most of the molecular structural parameters, including the H-bond parameters and the critical O=C–C–C torsion angle for the keto form were reproduced well by B3LYP/aug-cc-pvtz and MP2/cc-pvtz calculations.

Moriyasu *et al.*, [225] found an enol/keto ratio of 0.34 in 0.1 and 0.01 molar aqueous solutions at *T* = 298 K. Other experiments (for references, see Alagona *et al.* [232]) predict 0.14 as the lower limit. This suggests that although the enol form exists in aqueous solution, its fraction is much reduced even compared with its population at *T* = 671 K in the gas phase. On the basis of the experimental equilibrium compositions, the standard state free energy difference for the enol and keto forms is 2.7–4.9 kJ/mol. A number of recent theoretical in-solution studies have been performed for acetylacetone in order to reproduce this experimental value. Although any calculation predicted both stable enol and keto structures, their predicted ratio scattered considerably.

Ishida *et al.*, [233] succeeded to closely reproduce the lower limit of the experimental data by performing RISM-SCF calculations in water. The general reliability of the method is questionable, however, if considering that the predominant gas-phase structure was not correctly predicted and the prevailing tautomer was strongly overestimated in carbon tetrachloride. Schlund *et al.*, [234] calculated enol preference in aqueous solution and concluded that if the PCM model is used, the majority of the diketo form cannot be reproduced. Accordingly, Alagona *et al.*, [232] used the IEF-PCM method at different theoretical levels only for estimating the relative internal free energy. The relative solvation free energy was predicted by means of the MC/FEP method. The calculated best in-water total free energy difference was 0.7 kJ/mol, corresponding to enol/keto ratio of 0.75.

Related systems. Six-member rings with an intramolecular H-bond have been found for a number of systems such as 2-phenyliminomethyl-naphthalen-l-ol [235,236], its isomer, 1-phenyliminomethyl-naphthalen-2-ol, and substituted 3-hydroxy-4-pyridaldehyde deivatives [237]. For these molecules, the covalent structure assures the possibility of the intramolecular hydrogen bond in some preferable conformation. The authors concentrated, however, on the tautomeric issue, thus whether an N–H…O= or an =N…H–O hydrogen bond is more stable under the conditions in solution. These studies are mentioned here only as related systems, because the authors did not investigate the possible disruption of the intramolecular H-bond.

### 3.4. 7-Member Rings

With increasing molecular size, a smaller and smaller number of papers were found, which would deal with the maintenance of the intramolecular H-bond in different phases. This is not surprising because of the increasing difficulties in interpreting the experimental results for molecules with a longer aliphatic chain, or the exponential increase of the conformations, which would enormously increase the computer time even using medium size basis sets. Earlier in this paper, results of Chen *et al.*, [47] were referred to. The authors did not observe populations in the gas phase for 1,5-pentadiol conformations, which were stabilized by an intramolecular H-bond. Such a bond would involve formation of an eight-member ring. Thus this review ends here, where seven-member rings stabilized by a H-bond will be surveyed. Experimental gas-phase results have been found for γ-substituted carboxylic acids, although only for two of them.

#### γ-OH and γ-NH_2_ Carboxylic Acids

γ-OH butyric acid (GHB). The molecule is thermally unstable and converts to the cyclic butyrolactone structure by losing a water molecule. This feature makes the experimental investigation difficult. Regarding the gas-phase structure, only a conference abstract has been found by Suenram *et al.* [49]. By applying CP-FTMW (chirped pulse Fourier transform microwave) spectroscopy, the authors recorded the spectra of the α-, β-, and γ-OH butyric acids in order to study their conformational geometries and the effect of the internal H-bonding for the various compounds. The mixture for the γ-isomer did contain butyrolactone in the gas phase. The experimental results were compared with calculated MP2/6-311++G** + ZPE relative energies. For the γ-OH butyric acid, the attached slides give the impression that the molecule in its lowest-energy conformation forms an intramolecular H-bond between the alcohol OH and the carbonyl oxygen. The second lowest-energy conformer without an intramolecular H-bond is higher in energy by only 3 cm^−1^ (0.04 kJ/mol).

Nagy *et al.*, [48] studied eight selected conformations for γ-OH butyric acid up to the MP2/6-311++G** level. The lowest energy conformer was found to form an O–H…O= internal bond. In the second stable structure this bond is disrupted and the energy is higher by about 1 kJ/mol, but the calculated free energy difference at *T* = 310 K is more than 2 kJ/mol in favor of the structure without the indicated internal bond. It must be mentioned, however, that the thermal corrections were calculated at the HF/6-31G* level, and even their relative values may be exaggerated.

Using the gas-phase optimized geometries, the relative solvation free energies in aqueous solution were predicted for the eight conformers throughout MC/FEP simulations. The total free energy differed by 1.3 kJ/mol for the lowest and second-lowest stability species with extended aliphatic chains for each. No intramolecular H-bond exists even in the third-lowest free energy conformation of ΔG_tot_ = 2.3 kJ/mol. Thus, the water solvent disrupts the intramolecular H-bonds for γ-OH butyric acid and stabilizes extended conformations.

The solvent effect was studied in the same publication using mixed solvents of MeOH and CHCl_3_ in molar ratio of 2:1. The relative free energy is about 10 kJ/mol in favor of an extended structure in comparison with a nearly cyclic form prerequisite for the lactone formation. The calculated free energy difference is, however, relatively small as activation free energy for lactone formation in solution.

γ-NH_2_ butyric acid (GABA). The gas-phase conformation was studied by Blanco *et al.*, [50] using the LA-MB-FTMW technique. Five families including altogether nine conformations were observed in the experiment. Both fully extended conformations and those with NCCC gauche arrangement were identified. As mentioned, the two mostly populated species do not possess an intramolecular hydrogen bond.

Ramek and Nagy [238] studied the neutral/zwitterionic equilibrium of γ-NH_2_ butyric acid in aqueous solution. The total relative free energy was calculated as the sum of the internal free energy in the gas phase + relative solvation free energy. The zwitterion is not stable in the gas phase, thus its local-energy-minimum dihydrates were determined. For comparing the structures on equal footing, the dihydrates of the neutral conformers were optimized, as well. Among the isolated neutral species with geometries optimized in the dihydrate, the GABA structure without an intramolecular H-bond was found as the most stable conformation. The conformer with an intramolecular N…H–O–C=O bond was calculated to be higher in energy by 4.1 kJ/mol at the MP2/6-311++G**//HF/6-311++G** level.

The in-solution studies predicted the preference of each of the considered three zwitterionic forms relative to the neutral conformers. The most stable zwitterion had NCCC *gauche* and CCCC *trans* arrangements, thus the structure is fairly extended and exists without an intramolcular H-bond. Two cyclic zwitterionic conformers were investigated allowing for the formation of a –NH_3_^+^…^−^OCO bond. The extended form is more stable by at least 8.5 kJ/mol. No remarkable zwitterionic fraction was predicted, however, in slightly polar organic solvents such as chloroform and dicholoromethane. The partitioning between the aqueous and organic phases was predicted through the gradual shift of the zwitterionic to the neutral form in aqueous solution [239].

Crittenden *et al.*, [76] calculated the gas-phase conformational equilibrium and the relative stability of the neutral and zwitterionic GABA forms in solution. *Ab initio* (HF, MP2) and DFT (B3LYP) calculations using the 6-31G+* basis set were performed considering two and five explicit water molecules in a continuum dielectric solvent, modeled by COSMO. The most stable gas-phase structure out of nine studied conformers is an extended species without forming an intramolecular H-bond. In-solution studies proved the preference of the zwitteionic form, but if a pure solvent was placed into the cavity carved in the continuum water solvent, the preferred conformation for the zwitterion was a cyclic one with an intramolecular –NH_3_^+^…^−^OCO bond. No stable neutral GABA.2H_2_O structure was found, COSMO predicted different favorable zwitterionic structures. The extended structures are superior compared to the folded conformations. Both long-range and explicit water…GABA interactions preferentially stabilize the zwitterionic form. The authors allowed for the existence of a number of stable zwitterionic conformations in aqueous solution.

### 3.5. Acid-Base Complexes

For organic acid-base pairs, theoretical studies at satisfactorily high level convincingly prove that a neutral O–H…N bond rather than the ionic O^−^…^+^HN bridge is formed generally for complexes in the gas phase. Such calculations have been performed with acid components such as formic and acetic acids with various bases [115,116,240]. It has been found at the MP2/aug-cc-pvdz level, however, that the ionic acetic acid…methylguanidine complex is also stable on the gas-phase potential energy surface [115]. Investigations for further acid-base complexes is necessary whether a geometry optimization starting from an ion-pair structure could find a local energy minimum for this complex or the proton on the base moves onto the –COO^−^ group.

For an aqueous solution the question is, whether the intramolecular H-bond (from the perspective of the complex) saves its neutral-form character as in the gas phase or the ion-pair tautomer is stabilized. The answer could depend on the theoretical approach that has been applied for estimating the solvent effects. Liljefors and Orrby [240] concluded that consideration of an explicit water molecule would stabilize the ionic complex in continuum solvents with ε = 4–6. Without the explicitly considered water molecule, the ion-pair form is stabilized in continuum solvents with ε > 9.

Accordingly, Nagy and Erhardt [115] studied the possible proton jump within the complex using the IEF-PCM approach up to the CCSD(T)/CBS level and using MP2/aug-cc-pvdz and MP2/aug-cc-pvdz optimized geometries in solutions characterized by ε = 5 and 15. It was found that the acid …methylamine complexes are much more stable in the neutral than the ion-pair form. The complex is almost exclusively ionic, however, when the base is methylguanidine. This study aimed to explore the neutral or ionic character of the so-called salt-bridge formation when an Asp/Glu side chain can be close to the side chain of a Lys/Arg residue within a protein. The prediction was that the H-bond with lysine would be neutral in low-polarity environment, whereas the bridge is ionic with arginine in any environment. In partial disagreement with the above results, Silva *et al.*, [241] advocated the ionic character of any salt-bridge if an Asp side chain, as the acid partner is involved. These authors emphasized the need for considering more than three explicit water molecules and accounting for vibrational contributions to the total relative free energy even when a continuum solvent model is being applied. Preferably, consideration of large solvation shells was advised.

Nagy and Erhardt [116] studied the acetic acid interaction with methyl, dimethyl and trimethyl amines. The relative internal free energies were estimated using the IEF-PCM method at the *ab initio* CCSD(T)/CBS//MP2/aug-cc-pvdz and the DFT/B97D/aug-cc-pvtz levels. Relative solvation free energies were calculated through MC/FEP processes. The predicted total relative free energies varied considerably depending on the simulation conditions. When a solution of finite, about 0.1 molar concentration was modeled in the presence of a dissolved Na^+^Cl^−^ ion pair, the CH_3_COO^−^…^+^HNH*_x_*(CH_3_)_3−*x*_ (*x* = 0, 1, 2) ionic structure was found to predominate in aqueous solution. In contrast, when the low-dielectric-constant region of a protein was simulated by a solution model with chloroform solvent, the neutral form was found to be much more stable than the ion-pair. This is a remarkable conclusion regarding the type of the intermolecular H-bond for the complex, because the two modeling scenarios were supposed to account for the conditions of the drug-receptor interaction in an aqueous phase (e.g., on the surface of an enzyme) in comparison to a tightly binding environment within a cavity deeply buried in a protein.

## 4. Concluding Remarks

The focus of this review has been to compare gas-phase experimental structures where intramolecular hydrogen bonds (H-bonds) have been established, to in-solution theoretical calculations where experimental data are rarely available. The concept of the chemical bond has been the subject of a continuous research since the publication of the seminal paper of Lewis [242], and even recently a new chemical bond paradigm in terms of chemical action functional was published by Putz [243]. Intramolecular hydrogen bonds were assigned to structures on the basis of the 2011 IUPAC definition. A H-bond was defined as an attractive interaction in the form of (*X*)H…*Y*, where *X* is an atom more electronegative than H and *Y* has and electron pair which can favorably interact with the generally polar H. In addition to common heteroatoms, *Y* includes aromatic systems, whose electron clouds can interact with a polar hydrogen in the form of an H…*Y* π interaction. The source of the H-bond stabilization is mainly electrostatic and has remarkable contributions from a charge transfer from the acceptor to the donor. As a result, the H-bond has a partial covalent bond character between H and *Y*. The *X*–H…*Y* bond angle tends to be linear. It is important that no upper limit for the H…*Y* distance has been defined

The geometric parameters change only moderately for most systems upon dissolution if the conformation remains basically unaltered. The key parameters for intramolecular H-bonds are the (H)*X*CC*Y* torsional angles (*Y* = O, N, halogen in five-member rings or C in COOH and N in NO_2_ for six-member rings). This torsion angle can change remarkably upon dissolution in a solvent, leading to the separation of more than 300 pm for the *X* and *Y* atoms. In such cases, the intramolecular H-bond was considered in this review to have disrupted in solution. For intermolecular H-bonds, the *X*H and *Y* groups belong to two different molecules. An intermolecular H-bond was considered to be stable when the H…*Y* separation is 150–250 pm and there is a slightly bent *X*–H…*Y* moiety.

Intramolecular H-bonds were accepted for systems where the covalent structure allows for the formation of a five-, six- and seven-member ring including the polar hydrogen. This view was taken even if no (3, −1) BCP was found for the conformer in AIM analysis. Alternatively, structures with three- and four-member intramolecular rings were not considered as hydrogen-bonded species. The carboxylic group should be especially mentioned, where the larger stability of the *syn* O=C–O–H conformation in unsubstituted acids was not attributed to the existence of an intramolecular O–H…O= bond, but due to the electrostatic destabilization of the *anti-*conformation with facing electron pairs of the two oxygens.

Results of gas-phase experimental studies provide evidence for the existence of conformations favorable for an intramolecular H-bond with five- and six-member rings. Regarding seven-member rings, experiments indicate that the most stable conformations lack an intramolecular H-bond. Dissolution in slightly polar, non-protic organic solvents has little impact upon the gas-phase H-bond pattern. Indeed, these solvents are not competitors for forming a solute-solvent intermolecular H-bond *vs.* the intramolecular mode even when the solvent includes H-bond acceptor atoms such as in chlorinated methanes. Conformational equilibria of the enolic malondialdehyde could be affected, however, by a small amount of methanol in chloroform solution, where the methanol oxygen is a competitor for disrupting an intramolecular hydrogen bond. Non-protic solvents may also support the dimerization of solutes having two polar sites.

The theoretical challenge is the prediction of the H-bond pattern, intramolecular *vs.* intermolecular in aqueous (or some protic organic) solution. The survey generally shows that the structure found in the gas phase remains at least partially maintained in aqueous solution. Calculations mostly predict a shift in the conformer populations to allow for a larger population of the species with disrupted intramolecular H-bond. Appearance of the *X*CC*Y* *trans* conformation is a clear indication for this shift. Unfortunately, in-solution experiments do not provide quantitative information regarding the composition of the total *gauche* fraction or details for *ortho* phenols with rigid *X*CC*Y* skeletons where the polar hydrogen is not necessarily in an intramolecular bonding position. A remarkable exception is the group of aliphatic amino acids where studies have included the amino substituent in α, β, or γ position to the carboxylic group. All form zwitterions for which the intramolecular H-bond must then differ from its gas-phase counterpart. In contrast, the *ortho*-amino benzoic acid does not form a zwitterion in aqueous solution and maintains its gas-phase bonding pattern.

The calculated shift in the conformer population is a subtle problem. Examples show that the continuum dielectric solvent model may or may not lead to predictions in accord with the sparsely available experimental data. The basic conceptual problem is that this method, in its originally introduced version, does not account for the probably important solute-solvent intermolecular H-bond(s). Today, the supermolecule approach is widely used. It considers a small number of explicit solvent molecules surrounding the solute in a cavity carved in the dielectric solvent. Problems related to its application were discussed in detail. The main issue is that when a satisfactorily large number of solvent molecules are considered during the geometry optimization, the calculation is not practical. The convergence slows down enormously when a high-level theoretical method and a large basis set are applied. In the case of a DFT method, application of at least the aug-cc-pvtz basis set is needed for reliable estimation of relative internal free energies. The related frequency calculations then cause time-demanding calculations. An alternative for the calculation of the relative solvation free energy is the FEP method, which is also widely used with MC and MD simulations.

Polar structures like carboxylic acids or acid-base systems may form dimeric pairs/complexes in the gas phase. Another goal in this review to survey theoretical calculations that were performed to explore whether these structures are maintained in solution and if so, what protonation state will be stable in such complexes. Comparison of four theoretical and two experimental studies that followed the acetic acid dimerization, the obtained results are diverse. Molecular dynamics calculations, even performed by using the self-consistent charge *ab initio* method, predict no dimerization of this acid in aqueous solution. MD, however, provides changes in energies, not in free energies upon structural changes in the solution. In contrast, when integration of the pmf from MC and MD studies is undertaken so as to account for free energy changes, the predicted dimer fraction is 37%–45%. Calculated from the experimental dimerization constant, acetic acid could be dimerized by up to 14%.

The general solution for all of the computational problems mentioned above probably lies in using *ab initio* molecular dynamics modeling. This should allow for the application of a high-level method (e.g., CCSD(T)), large basis set (aug-cc-pvtz) and flexible molecular geometry for all participants of the system. Simulations having a few hundred solvent molecules, which is necessary for a reasonable modeling of the solution, and a production phase of tens of ns are not affordable today. However, the rapidly increasing capacity of computers and ongoing production of more efficient programs will likely allow performing such calculations in the not too far future. Despite attractive features of the Car-Parrinello method [107], the required computer time is still too large for routine use of the method for in-solution calculations. Nonetheless, QM/MM [106] and self-consistent charge density-functional based tight-binding [131] methods applying at least medium-size basis set can be used even today for solving the problem addressed in the title of this review.

If none of these suggestions is practical for following a specific structural problem, MD is recommended for solution structure simulations provided care is taken to establish a proper parameterization of the force field. The latter is not trivial, however, for molecules with possible intramolecular *vs.* intermolecular H-bonds. For calculating conformer/tautomer equilibria, this author recommends the supermolecule approach in a continuum solvent for geometry optimization and relative internal free energy calculation at high theoretical level while including a satisfactorily large number of solvent molecules. Finally, in the case of a protic solvent, MC/FEP and MD/FEP methods are recommended for relative solvation free energy calculations.
